# Combating Drug Resistance in *Mycobacterium Tuberculosis*: A Combinatorial in Silico and Experimental Modeling Approach Toward Novel ATP Synthase Inhibitor Discovery

**DOI:** 10.1177/11779322261438313

**Published:** 2026-04-08

**Authors:** Haidy H. El-Zoheiry, Ratul Bhowmik, Ajay Manaithiya, Rajarshi Ray, Mahesh Samantaray, Amutha Ramaswamy, Ashok Aspatwar

**Affiliations:** 1 Department of Pharmaceutical Chemistry, Faculty of Pharmacy, Cairo University, Cairo, Egypt; 2 Faculty of Medicine and Health Technology, Tampere University, Tampere, Finland; 3 Department of Translational Medicine, All India Institute of Medical Sciences (AIIMS), Bhopal, Bhopal, India; 4 Department of Bioinformatics, School of Life Sciences, Pondicherry University, Puducherry, India

**Keywords:** Structure-based pharmacophore modeling, machine learning, QSAR, molecular docking, molecular dynamics simulations, antimycobacterial inhibition assay, toxicity studies

## Abstract

Adenosine triphosphate (ATP) synthase in *Mycobacterium tuberculosis* (Mtb) is essential for energy metabolism through oxidative phosphorylation, where ATP is synthesized from ADP. This enzyme supports bacterial survival during both active growth and dormancy, enabling Mtb to persist under stressful conditions. During dormancy, Mtb enters a non-replicating, drug-tolerant state that reduces the effectiveness of many antibiotics. Inhibition of ATP synthase therefore disrupts ATP-dependent survival mechanisms in Mtb. Although this target has been clinically validated by bedaquiline, the emergence of resistance and the limited chemical diversity of reported inhibitors indicate a clear need for new ATP synthase-targeting compounds. In this study, we employed an integrative pipeline combining structure-based pharmacophore modeling, artificial neural network-driven quantitative structure-activity relationship (ANN-QSAR) modeling, and absorption distribution metabolism excretion and toxicity (ADMET)-based pharmacokinetic filtering strategies to screen an antituberculosis-targeted library of approximately 4200 molecules from the Life Chemicals database. Initial screening identified 8 hit molecules characterized by key molecular features previously highlighted as positive contributors in both Shapley Additive Explanations (SHAP) and Pearson correlation analyses, including SubFP1 (primary carbon), SubFP88 (carboxylic acid derivative), SubFP143 (carbonic acid derivative), SubFP9 (alkyl fluoride), SubFP137 (vinylogous ester), SubFP184 (heteroaromatic), SubFP26 (tertiary aliphatic amine), and SubFP171 (aryl chloride). Molecular docking and molecular dynamics simulation studies (200 ns) further highlighted molecules F0526-1306 and F0526-1309 as the most promising candidates. Subsequent antimycobacterial inhibition assays demonstrated that both molecules significantly reduced mycobacterial biofilm formation. In addition, toxicity evaluations using a zebrafish model confirmed the safety and favorable tolerability of these molecules, supporting their potential as viable candidates for further preclinical and in vivo drug development studies.

## Graphical abstract

**Graphical Abstract:**
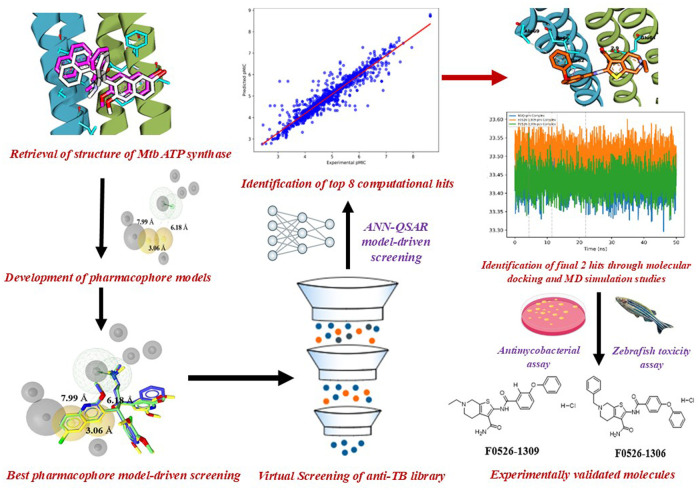

## Introduction

A defining feature that sets *Mycobacterium tuberculosis* (Mtb) apart from many other bacterial pathogens is its ability to switch between active and dormant states in response to stressful conditions.^1^ In 2022, Mtb was ranked as the second leading cause of death globally among single infectious agents.^2^ The global incidence of tuberculosis is approximately 10 million cases per year, with a mortality rate of about 50% in the absence of treatment and approximately 15% with treatment following World Health Organization guidelines.^2^ In its dormant state, Mtb transitions into a non-replicating, drug-tolerant form characterized by minimal metabolic activity.^3^ To sustain dormancy, the bacilli rely on low-level energy production, which is primarily orchestrated by adenosine triphosphate (ATP) synthase.^4^

Adenosine triphosphate synthase is the key molecular machinery responsible for energy production in Mtb, as well as in all living organisms.^4^ It functions as a molecular engine that generates ATP, the universal cellular energy currency, through oxidative phosphorylation of adenosine diphosphate (ADP). The mycobacterial F1F0-ATP synthase consists of a membrane-embedded F0 domain and a cytoplasm-facing F1 domain. The F0 domain comprises subunits a and b along with a ring formed by 9 c subunits, whereas the F1 domain is composed of the alpha3beta3 catalytic headpiece and the gamma, delta, and epsilon subunits. The gamma and epsilon subunits, together with subunit b, form the central and peripheral stalks that mechanically connect the F0 c-ring to the F1 catalytic core.^5^

Bedaquiline is the first clinically approved inhibitor of mycobacterial ATP synthase and has demonstrated that inhibition of bacterial energy production is effective against both replicating and dormant Mtb. The drug exerts its activity by binding to the c subunit of ATP synthase. However, due to its high lipophilicity, bedaquiline exhibits a prolonged terminal half-life and is associated with cardiotoxicity, particularly QT interval prolongation. These limitations underscore the need to develop new ATP synthase inhibitors with reduced lipophilicity and improved safety profiles.^6^ Collectively, these observations establish ATP synthase as a promising therapeutic target for combating both active and dormant Mtb. Several next-generation and experimental ATP synthase inhibitors have been reported, including diarylquinoline derivatives and alternative chemotypes such as squaramides and γ-loop-targeting molecules.^7-9^ Nevertheless, the chemical diversity explored to date remains limited, and many reported compounds exhibit safety concerns or diminished efficacy due to resistance, highlighting the need for novel and structurally diverse ATP synthase inhibitors supported by computational screening and early biological evaluation.

Computational approaches, including quantitative structure-activity relationship (QSAR) modeling, pharmacophore modeling, molecular docking, and molecular dynamics (MD) simulations, play a critical role in modern drug discovery by facilitating the identification and optimization of compounds with improved therapeutic potential. Cheminformatics and molecular modeling techniques have been widely applied for decades to support drug discovery and lead optimization across multiple therapeutic areas. Today, in silico modeling constitutes an integral component of the early stages of the drug development pipeline, enabling efficient identification of promising candidates prior to extensive experimental validation.^10^ In this study, we present a comprehensive computational framework for the rational design of novel Mtb ATP synthase inhibitors targeting the c subunit, integrating structure-based pharmacophore modeling, machine learning-driven quantitative structure-activity relationship (ML-QSAR) modeling, virtual screening, and MD simulations. In addition, antimycobacterial and zebrafish-based toxicity assays were performed on the top computational hits to experimentally validate the predictive power and novelty of the computational approach.

## Materials and Methods

### Pharmacophore model

#### Data set and pharmacophore modeling

A receptor-based shared-feature pharmacophore model was developed using LigandScout v4.5 (https://www.inteligand.com/ligandscout/).^11^ Three protein structures (PDB IDs: 8J57, 8JR1, and 8G0B), representing mycobacterial ATP synthase co-crystallized with bedaquiline, TBAJ-587, and TBAJ-876, respectively, were retrieved from the Protein Data Bank (https://www.rcsb.org/) (Table 1).^12,13^ Initially, individual structure-based pharmacophore models were generated separately for each PDB structure. These models were subsequently transferred to the “Alignment Perspective” window to ensure proper structural alignment and to identify shared pharmacophoric features using the “Generate Shared Feature Pharmacophore” tool. Excluded volume features were incorporated to define the steric constraints of the binding sites. As a result, 10 3-dimensional pharmacophore models were generated, differing in the number of excluded volumes, feature weights, and tolerance settings (Table 2).

**Table 1.** The selected PDB files for pharmacophore modeling.

PDB ID	Co-crystallized ligand	Ligand name	2D interactions	Reference
8J57	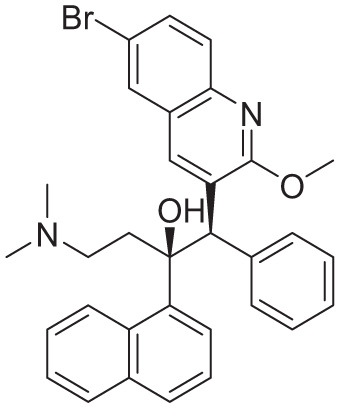	Bedaquiline	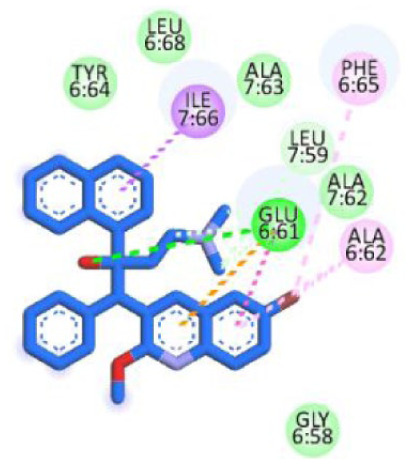	Zhang et al^12^
8JR1	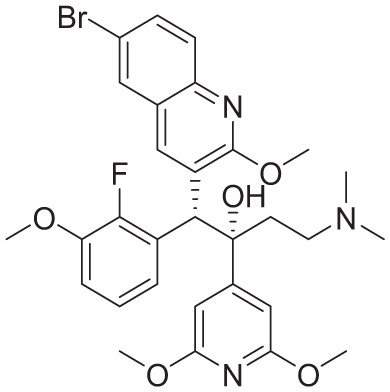	TBAJ-587	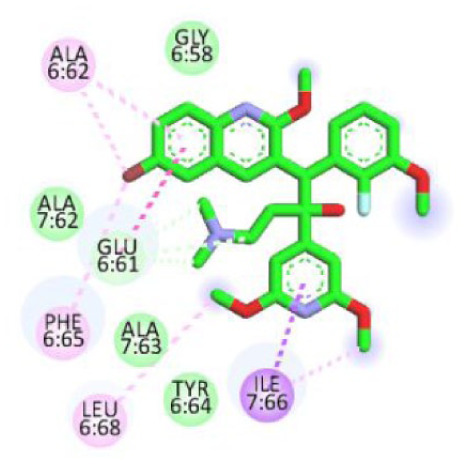	Zhang et al^12^
8G0B	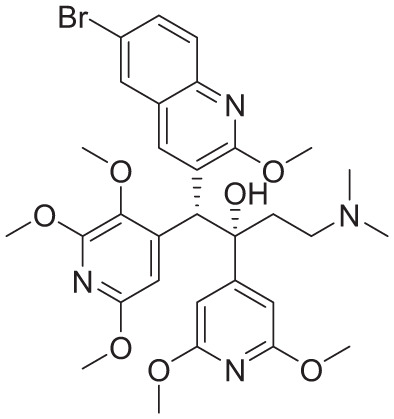	TBAJ-876	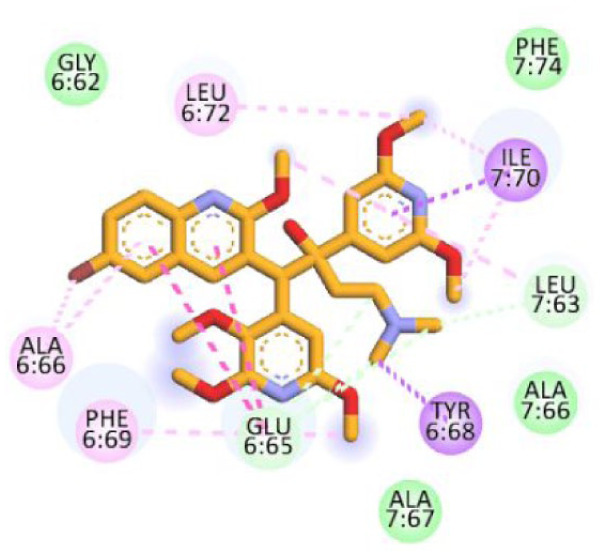	Courbon et al^13^

**Table 2.** Brief of the pharmacophoric features of the generated models.

Ph4 model	No. of features	F1	F2	F3	Number of excluded volumes
Ph4-1	10	Hyd.	Hyd.	Don.	7
Ph4-2	12	Hyd.	Hyd.	Don.	9
Ph4-3	9	Hyd.	Hyd.	Don.	6
Ph4-4	11	Hyd.	Hyd.	Don.	8
Ph4-5	11	Hyd.	Hyd.	Don.	8
Ph4-6	12	Hyd.	Hyd.	Don.	9
Ph4-7	10	Hyd.	Hyd.	Don.	7
Ph4-8	11	Hyd.	Hyd.	Don.	8
Ph4-9	11	Hyd.	Hyd.	Don.	8
Ph4-10	11	Hyd.	Hyd.	Don.	8

Hyd. = hydrophobic feature, Don. = donor feature.

#### Pharmacophore model selection and validation

To select and validate the best-performing pharmacophore model, a test set comprising 710 molecules was employed. This set included 20 structurally diverse active compounds, self-collected from the literature (Supplementary Table S1), along with 690 inactive decoys generated using the DUDE-Z platform (https://tldr.docking.org/start/dudez). The self-collected active molecules were selected based on their reported high inhibitory activity and relatively low lipophilicity, with the aim of mitigating the known side effects associated with bedaquiline. Maximum chemical diversity was ensured by selecting the most potent representative compound(s) from each published study.

**Figure 1.**
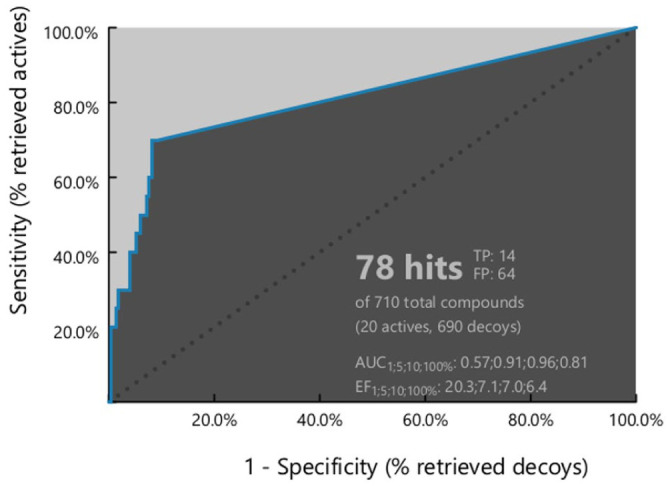
ROC curve with AUC value for the elected pharmacophoric model “Ph4-7.”

For each pharmacophore model, the numbers of true positives (TP), false positives (FP), true negatives (TNs), and false negatives (FNs) were determined. These values were subsequently used to calculate a comprehensive set of evaluation metrics, including sensitivity (Se), specificity (Sp), yield of actives (Ya), enrichment factor (E), accuracy (Acc), discrimination ratio (DR), F1 score (F1), and the Matthew correlation coefficient (MCC) (Supplementary Table S2 and Table 3). Other metrics were calculated as the receiver operating characteristic (ROC) and its area under the curve (AUC) (Figure 1).

**Table 3.** The performance metrics of the assembled models, the selected one is bolded, on the test set (active compounds and decoys).

Ph4 model	N	TP	TN	A	n	FP	FN	Se	Sp	Ya	E	Acc	DR	F1	MCC
Ph4-1	710	13	651	20	72	59	7	0.65	0.943	0.181	6.410	0.935	0.689	0.283	0.310
Ph4-2	710	11	627	20	94	83	9	0.55	0.909	0.117	4.154	0.899	0.605	0.193	0.211
Ph4-3	710	8	618	20	100	92	12	0.40	0.896	0.080	2.840	0.882	0.447	0.133	0.128
Ph4-4	710	11	641	20	80	69	9	0.55	0.929	0.138	4.881	0.918	0.592	0.220	0.237
Ph4-5	710	12	640	20	82	70	8	0.60	0.928	0.146	5.195	0.918	0.647	0.235	0.259
Ph4-6	710	10	620	20	100	90	10	0.50	0.899	0.100	3.550	0.887	0.556	0.167	0.177
**Ph4-7**	**710**	**14**	**646**	**20**	**78**	**64**	**6**	**0.70**	**0.936**	**0.179**	**6.372**	**0.930**	**0.748**	**0.286**	**0.322**
Ph4-8	710	11	599	20	122	111	9	0.55	0.868	0.090	3.201	0.859	0.634	0.155	0.172
Ph4-9	710	14	571	20	153	139	6	0.70	0.828	0.092	3.248	0.824	0.846	0.162	0.202
Ph4-10	710	13	599	20	124	111	7	0.65	0.868	0.105	3.722	0.862	0.749	0.181	0.215

N = the total number of compounds in the data set, TP = true positive, TN = true negative, A = the number of actives, n = hits count, FP = false positive, FN = false negative, Se = selectivity, Sp = specificity, Ya = yield of actives, E = enrichment, Acc = accuracy, DR = discrimination ratio, F1 = F1 Score, MCC = Matthew correlation coefficient.

#### Pharmacophore-based virtual screening and hit filtration

The best-designated pharmacophore model, Ph4-7, was integrated into the virtual screening workflow using LigandScout v4.5. Screening was performed on the *Anti-tuberculosis Focused Library* obtained from Life Chemicals (https://lifechemicals.com/screening-libraries/targeted-and-focused-screening-libraries/antituberculosis-library). This library comprises 4200 drug-like screening compounds targeting key proteins involved in anti-tuberculosis drug discovery. LigandScout pre-maps libraries into pre-calculated conformational ensembles prior to initiating the virtual screening process. Hit compounds were identified using the *Pharmacophore Fit* scoring function, with the options “Match All Query Features” and “Get Best Matching Conformation” enabled, while accounting for excluded volume constraints. The ChemBioServer platform (https://chembioserver.vi-seem.eu/simple_search.php) was used to filter the retrieved hits from the virtual screening step.^14^ Using the “Predefined Queries” filtering approach, Lipinski, Veber, and Ghose filters were applied to the hit molecules. Finally, the False Positive Remover platform (https://www.cbligand.org/PAINS/login.php) was employed to further eliminate PAINS-containing compounds.

#### Structure-activity relationship analysis

To elucidate mechanistic insights into the inhibition mechanism through structure-activity relationship (SAR) analysis, a data set of 17 Mtb ATP synthase inhibitors was retrieved from the ChEMBL database (https://chembl.gitbook.io/chembl-interface-documentation/web-services).^15^ Chemical structures were obtained as Simplified Molecular Input Line Entry System (SMILES) representations, and biological activity data were collected as half-maximal inhibitory concentration (IC_50_) values. Data curation was performed using the ChEMBL web resource client Python module.

To ensure a normalized scale for SAR analysis and to reduce variability in the data, compounds were categorized as highly active (IC_50_ < 100 nM) or weakly active (IC_50_ ≥ 100 nM). Substructure fingerprints (n = 307) were calculated for the 17 inhibitors using *padelpy* (https://github.com/ecrl/padelpy), a Python wrapper for the PaDEL-Descriptor software.^16^ Subsequently, the *pandas* (https://pypi.org/project/streamlit-pandas/), *seaborn* (https://pypi.org/project/seaborn/), and *matplotlib* (https://matplotlib.org/) Python packages were used to compute the mean presence of each substructure fingerprint across activity classes. Only features with a mean presence greater than 0.4 in at least 1 activity class were retained for visualization. A heatmap was generated to highlight activity class–specific patterns in molecular fingerprints.

#### Quantitative structure-activity relationship data set generation and modeling

##### Data set generation and machine learning-driven quantitative structure-activity relationship model development

We retrieved 2168 molecules that had been experimentally tested in antimycobacterial/antitubercular assays from the ChEMBL database. Substructure fingerprints (n = 307) were calculated for these molecules using the *padelpy* Python wrapper. To identify structurally similar molecules with potential Mtb ATP synthase inhibitory activity, a Tanimoto similarity coefficient threshold of 40% was applied by mapping 17 previously curated Mtb ATP synthase inhibitors onto the larger anti-TB data set of 2168 molecules based on substructure fingerprints.

The top 100 molecular features were subsequently identified using variance importance plots (VIP) for both the 17 curated ATP synthase inhibitors and the 40% Tanimoto similarity–based subset derived from the 2168 molecules. Similarities between the top 100 features of the curated inhibitors and the similarity-based subset were visualized using the Venn diagrams. In addition, Uniform Manifold Approximation and Projection (UMAP) plots were generated using molecular fingerprint data to visualize chemical similarity in 2-dimensional space for both data sets. Overall, this approach aimed to curate a relevant QSAR data set from the antimycobacterial assay data that exhibited at least 40% structural similarity to the previously curated ATP synthase inhibitors.

Following curation of the hypothetical data set for QSAR modeling of ATP synthase inhibition activity, multiple feature selection methods were applied. Variance thresholding and Pearson correlation–based selection (correlation coefficient > 0.9) were used to remove constant and highly correlated features, respectively, from the previously calculated substructure fingerprints. To normalize the response variable and reduce variability in the ML-QSAR analysis, minimum inhibitory concentration (MIC) values for 1715 molecules were converted to predicted minimum inhibitory concentration (pMIC) values, defined as the negative base-10 logarithm of MIC. The data set was then divided into training and test sets using an 80:20 split ratio.

Support vector machine (SVM), artificial neural network (ANN), random forest (RF), and XGBoost algorithms were implemented to develop robust QSAR models by combining substructure and PubChem fingerprint data sets using the *scikit-learn* Python library (https://github.com/RatulChemoinformatics/QSAR-Models). For the SVM model, a radial basis function (RBF) kernel was employed to capture non-linear relationships between molecular fingerprints and biological activity, with optimization of the C (regularization) and gamma (kernel coefficient) parameters. For the ANN-based QSAR model, a feedforward neural network (FNN) with a single hidden layer was implemented, and the number of neurons, activation functions (ReLU), and optimizer (Adam) were optimized. In the RF-based QSAR model, an ensemble of decision trees combined with feature bagging was used to enhance predictive performance, with optimization of the number of estimators, tree depth, and minimum samples per split. For the XGBoost-based QSAR model, key hyperparameters—including the number of estimators, tree depth, learning rate, subsample ratio, and feature selection ratio—were optimized to improve model robustness and predictive accuracy.

Following initial model development, principal component analysis (PCA) was performed to assess compound distribution and identify potential outliers in both the training and test data sets. The PCA was used to project high-dimensional descriptor space into a lower-dimensional space while retaining maximal variance. The first 2 principal components were visualized using scatter plots to examine clustering patterns and identify data points deviating from the main distribution. Molecules falling outside the primary clusters were considered outliers and were removed from further modeling. After outlier removal, the models were retrained using grid-based hyperparameter tuning and cross-validation with SVM, ANN, XGBoost, and RF algorithms.

To identify the best-performing QSAR model, multiple statistical metrics were calculated, including root mean squared error (RMSE), mean squared error (MSE), mean absolute error (MAE), Pearson correlation coefficient, and 10-fold cross-validation metrics. The model exhibiting the lowest RMSE, MSE, and MAE values, while maintaining a high Pearson correlation coefficient and strong cross-validation performance, was selected as the optimal QSAR model for predicting ATP synthase inhibition activity. The selected model was subsequently used to predict the biological activity of computational hits identified through pharmacophore- and absorption distribution metabolism excretion and toxicity (ADMET)-based screening.

#### Feature elucidation of the machine learning-driven quantitative structure-activity relationship model for rational drug design

To further enhance the interpretability of the final ML-QSAR model, multiple feature importance analysis techniques were employed, including Variance Importance in Projection (VIP) analysis, Pearson correlation plot analysis, and Shapley Additive Explanations (SHAP) analysis. For VIP analysis (https://scikit-learn.org/stable/api/sklearn.feature_selection.html), Partial Least Squares Regression (PLSR) was used to rank molecular fingerprints based on their contribution to the model.

For Pearson correlation plot analysis (https://scikit-learn.org/stable/modules/generated/sklearn.feature_selection.r_regression.html), a pairwise correlation matrix was generated to identify positively and negatively correlated molecular fingerprints across the models. For SHAP analysis (https://shap.readthedocs.io/en/latest/), SHAP values were calculated for each fingerprint to interpret the influence of individual molecular features on pMIC values across the data set.

In addition, clustering analysis was performed for highly active (pMIC ≥ 8) and weakly active (pMIC ≤ 3) molecules using the Tanimoto coefficient–based clustering (https://github.com/MunibaFaiza/tanimoto_similarities). This clustering approach enabled the identification of shared molecular features among compounds exhibiting strong or weak inhibitory activity. To further improve interpretability, a WordCloud-based visualization (https://amueller.github.io/word_cloud/) was employed to highlight the most frequently occurring molecular features within each activity group.

#### Molecular docking study

The 3-dimensional crystal structure of Mtb ATP synthase (PDB ID: 8J57) was retrieved from the Protein Data Bank (https://www.rcsb.org/).^17^ Prior to docking, water molecules and non-essential chains were removed, retaining only chains 6 and 7. The protein structure, along with the 8 hit candidate molecules, was preprocessed using the *Dock Prep* tool in UCSF Chimera v1.17.3 for validation and docking.^18^ This preprocessing step included the addition of hydrogen atoms, assignment of Gasteiger charges, and energy minimization using steepest-descent and conjugate gradient algorithms.

A validation step was initially performed to confirm the suitability of the selected docking protocol. Molecular docking was carried out using AutoDock Vina with the number of iterations set to 100.^19^ The docking grid box was defined based on the coordinates of the co-crystallized ligand present in the PDB structure. Molecular interactions, including hydrogen bonding, hydrophobic contacts, and van der Waals interactions, were analyzed using Discovery Studio Visualizer 2021 (https://www.3ds.com/products/biovia/discovery-studio/visualization).

#### Molecular dynamics simulation and post-dynamic binding free energy analysis

Molecular dynamics simulations were carried out for bedaquiline, F0526-1309, and F0526-1306 with Mtb ATP synthase complexes using all-atom MD simulations with GROMACS 2022.6, employing the AMBER99SB-ILDN force field.^20,21^ Ligand topology parameters were generated using the ACPYPE webserver.^22^ The systems were further solvated using the TIP3P water model and a cubic box type with a minimum distance of 1.0 nm between the solute and box edge. Sodium ions were added to neutralize the system. The steepest-descent algorithm was used to optimize the energy of the system with a maximum tolerance of 1000 kJ/mol/nm.^23^ Subsequently, all the systems were equilibrated using a Berendsen thermostat (V-rescale) and Parrinello-Rahman pressure coupling algorithm at 300K and 1 Pascal bar pressure, respectively.^24,25^ All 3 complexes were equilibrated for a period of 100 ps under constant Number of particles, Volume, and Temperature (NVT) and constant Number of particles, Pressure, and Temperature (NPT) conditions. Finally, MD simulations of 200 ns were performed for all 3 complexes. The information from the entire trajectory was used to analyze the root mean square deviation (RMSD) of backbone atoms, root mean square fluctuation (RMSF), radius of gyration (Rg), and solvent-accessible surface area (SASA) and the number of Hydrogen Bonds (HBond) using the Xmgrace tool. The g_mmpbsa module was used to calculate the binding free energy (ΔG) of the 3 complexes using the trajectories of the complexes simulated for a period of 200 ns.^26,27^

#### Validation of computational hits using in vitro inhibition of *Mycobacterium marinum*

The top 2 hit molecules, F0526-1306 and F0526-1309, identified through computational screening, were procured from Life Chemicals, Inc (Burlington, Ontario, Canada; https://lifechemicals.com/). For *Mycobacterium marinum* inhibition studies, each compound was dissolved in dimethyl sulfoxide (DMSO) to prepare 100 mM stock solutions. Serial dilutions were subsequently prepared in distilled water and used for inhibition assays in 96-well microtiter plates.

A bioluminescent *M marinum* strain (ATCC 927; Mmr) expressing the pMV306hsp + LuxG13 cassette was used for the inhibition studies. The pMV306hsp + LuxG13 plasmid was a generous gift from Brian Robertson and Siouxsie Wiles (Addgene plasmid #26161; http://n2t.net/addgene:26161; RRID: Addgene_26161).^28^ Bacteria were cultured on Middlebrook 7H10 agar (Becton, Dickinson and Company, Franklin Lakes, New Jersey) supplemented with 10% (v/v) oleic acid-dextrose-catalase (BD BBL™ Middlebrook OADC Enrichment) and 0.5% (v/v) glycerol at 29°C in the dark for 7 days. For biofilm-based inhibition assays, cultures were inoculated into a biofilm-promoting medium consisting of Middlebrook 7H9 broth (Sigma-Aldrich, St. Louis, Missouri) supplemented with 10% (v/v) albumin-dextrose-catalase (BD BBL™ Middlebrook ADC Enrichment). A bacterial suspension adjusted to an optical density at 600 nm (OD_600_) of 0.1 was aliquoted into sterile white 96-well microtiter plates (184 µL per well; PerkinElmer, Waltham, Massachusetts). The plates were sealed with Parafilm M (Bemis) and incubated in the dark at 29°C for 7 days.

#### Positive control and comparator treatments

Rifampicin (RIF) was included as a positive control for antimycobacterial growth inhibition to confirm assay performance and as a comparator in combination experiments. The RIF was used at a fixed concentration across all experiments (400 µg/mL), while the test compounds were evaluated over a concentration range of 20, 50, 100, 200, and 400 µM. Because rifampicin inhibits bacterial RNA polymerase rather than ATP synthase, it was employed as a growth-inhibition reference control and combination partner, not as a mechanistic ATP synthase inhibitor control. The UNT denotes untreated bacterial cultures (no compound). Where applicable, vehicle controls contained the same final DMSO concentration as treatment wells.

#### Toxicity studies of computational hits using zebrafish models

For toxicity evaluation, the compounds F0526-1306 and F0526-1309 were dissolved in DMSO (Sigma-Aldrich) to prepare 100 mM stock solutions, which were stored at −20°C until use. Prior to each experiment, serial dilutions were prepared from the stock solutions using embryonic medium composed of 5.0 mM NaCl, 0.17 mM KCl, 0.33 mM CaCl_2_, 0.33 mM MgSO_4_, and 0.1% (w/v) methylene blue (Sigma-Aldrich), following previously established protocols.^29,30^ Zebrafish embryos at 1 day post-fertilization (dpf) were exposed to the diluted inhibitor solutions for toxicity assessment.

Wild-type AB strain adult zebrafish were maintained in an incubator at 28.5°C. For embryo collection, 3 to 5 breeding pairs were set up overnight. The following morning, embryos were collected 1 to 2 hours post-fertilization (hpf) using a sieve and rinsed thoroughly with embryonic medium consisting of 5.0 mM NaCl, 0.17 mM KCl, 0.33 mM CaCl_2_, 0.33 mM MgSO_4_, and 0.1% (w/v) methylene blue (Sigma-Aldrich). Embryos were then maintained at 28.5°C overnight, and toxicity evaluation was performed at 24 hpf. All zebrafish experiments were conducted at the Zebrafish Core Facility, Tampere University, Finland, following protocols established in our laboratory.^30^

To assess compound toxicity, 1-dpf zebrafish embryos were exposed to 4 different concentrations of each inhibitor, with 20 larvae used per concentration.^30^ Inhibitor concentrations ranged from 25 µM to 400 µM. Control groups consisted of equal numbers of untreated larvae and larvae treated with 1% DMSO. Toxicity assays were performed in 24-well plates (Corning Costar cell culture plates), with 3 to 4 embryos placed per well containing 1 mL of embryonic medium supplemented with the respective inhibitor concentration. Larval survival was monitored every 24 hours for 5 days post-exposure.

After 5 days of exposure, toxicity effects were evaluated by examining 8 phenotypic parameters: (1) hatching, (2) edema, (3) movement pattern, (4) yolk sac utilization, (5) heartbeat, (6) body shape, (7) swim bladder development, and (8) otolith sac development. Observations were recorded for each treatment group using a stereomicroscope. Images of developing larvae were captured using a stereomicroscope equipped with a camera, following the standardized laboratory protocol for toxicity and safety assessment of computational hit molecules.^30^

#### Prediction of molecular mechanisms of final hit molecules at the host-pathogen interface

To further explore the immunomodulatory mechanisms of action of the top hit molecules under tuberculosis conditions at the host-pathogen interface, a network pharmacology and systems biology approach was employed. This approach involved target prediction for the final lead molecules, followed by tuberculosis-associated gene mapping, pathway enrichment and gene ontology (GO) analysis, and single-cell RNA expression analysis using multiple data analysis strategies.

Initially, the SwissTargetPrediction server (https://www.expasy.org/resources/swisstargetprediction) was used to predict potential targets of the individual molecules based on their SMILES representations.^31^ Protein-protein interaction data for the predicted targets were then curated from the STRING database (https://string-db.org/). The degree method implemented in the cytoHubba plugin (https://apps.cytoscape.org/apps/cytohubba) within Cytoscape software (https://cytoscape.org/) was used to identify the top 5 hub genes associated with each molecule.^32-34^ Subsequently, a Venn diagram–based analysis (https://bioinfogp.cnb.csic.es/tools/venny/index2.0.2.html) was performed to identify overlapping hub genes between the predicted molecular targets and a tuberculosis-associated gene data set retrieved from the Comparative Toxicogenomics Database (CTD) (https://ctdbase.org/).^35^

The overlapping genes identified from the Venn analysis were subjected to GO and pathway enrichment analysis using the Enrichr-KG platform (https://maayanlab.cloud/enrichr-kg).^36^ In addition, cell-type–specific expression of the identified hub genes was examined using single-cell RNA-sequencing (scRNA-seq) data from an Mtb granuloma data set available through the Broad Institute Single Cell Portal (https://singlecell.broadinstitute.org/single_cell/study/SCP1749/cellular-ecology-of-m-tuberculosis-granulomas-4-week-dataset).

Single-cell RNA-sequencing analysis was performed using the Seurat package (https://satijalab.org/seurat/). The data set underwent normalization, identification of variable features, and scaling, with highly variable genes visualized using feature plots. Principal component analysis was conducted for dimensionality reduction, supported by elbow plots and heatmaps to select optimal principal components. Clustering analysis was then performed based on the selected principal components, followed by UMAP visualization to annotate distinct cell-type clusters. Differential gene expression (DEG) analysis was conducted using the Wilcoxon rank-sum test to identify marker genes for each cluster. Finally, expression levels of hub genes associated with the individual hit molecules were analyzed across specific cell types using feature plots, dot plots, and violin plots (https://github.com/RajarshiRay25/Single-Cell-Analysis—Tuberculosis).

#### Ethics statement

All zebrafish experiments were performed at the zebrafish core facility, University of Tampere. Experiments were conducted using zebrafish larvae younger than 7 dpf, which do not require project-specific ethical approval under applicable national regulations.^29^ The facility holds an establishment authorization granted by the National Animal Experiment Board (ESAVI/7975/04.10.05/2016). All procedures were carried out in accordance with institutional guidelines to minimize animal stress and ensure humane treatment. The reporting of this study conforms to the ARRIVE guidelines for reporting animal research (see Supplementary File Author Checklist—E10 only).

## Results and Discussion

### Pharmacophore model

Using the shared-feature structure-based pharmacophore design approach implemented in LigandScout v4.5, a total of 10 distinct pharmacophore models were generated. Although the parameter values of Ph4-1 and Ph4-7 were comparable, Ph4-7 was selected as the best-performing model due to its superior sensitivity and discrimination ratio (Table 3). Ph4-7 comprises 3 pharmacophoric features, including 2 hydrophobic features and 1 hydrogen bond donor feature with its corresponding projection point, along with 7 exclusion volumes distributed around the active site (Supplementary Figure S1).

Ph4-7 demonstrated its ability to discriminate active molecules from decoys by identifying a total of 78 hits, including 14 TPs and 64 FPs. The model achieved a sensitivity of 0.70, correctly identifying 14 out of 20 active compounds. In addition, it exhibited a high specificity of 0.936 by correctly excluding 646 TNs from 690 decoy compounds. The model also displayed a yield value of 0.179 and an enrichment factor of 6.372, along with accuracy and discrimination ratio values of 0.930 and 0.748, respectively.

The ROC curve showed an initial sharp increase followed by a plateau, indicating the model’s ability to prioritize active compounds over inactive ones. Furthermore, an AUC value of 0.81 suggests that the model functions as a moderate to excellent classifier.

Virtual screening of the *Anti-tuberculosis Focused Library* resulted in the identification of 1167 hit compounds. Following the application of the “Predefined Queries” and “Toxicophores” filters using the ChemBioServer platform, 12 and subsequently 9 compounds were identified as drug-like hits. Final refinement using the False Positive Remover platform yielded 8 PAINS-free hit compounds. The fitting of the final molecules to the selected pharmacophore model is illustrated in Table 4 and Supplementary Figure S2.

**Table 4.** The final hits retrieved after virtual screening, drug-like property, and PAINS filtering.

No	Hit structure	No	Hit structure
1	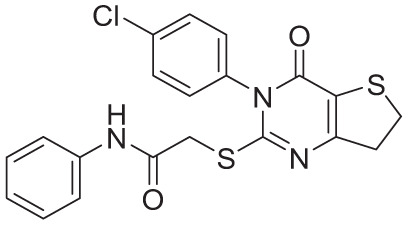 F0579-0616	2	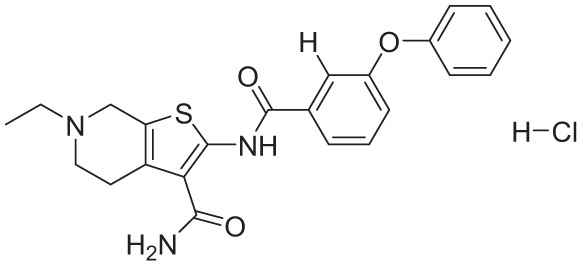 F0526-1309
3	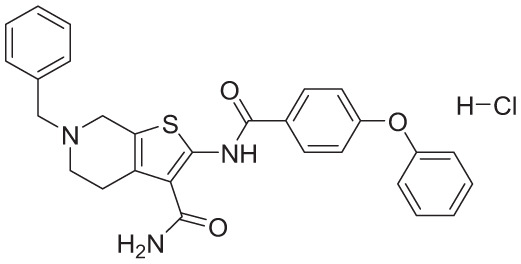 F0526-1306	4	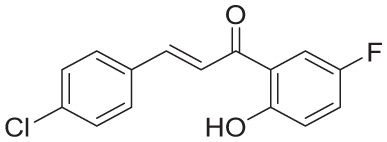 F1190-0509
5	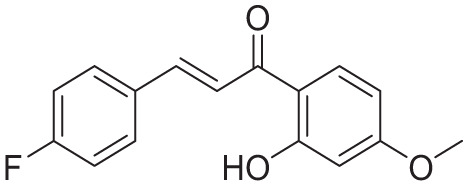 F1190-0598	6	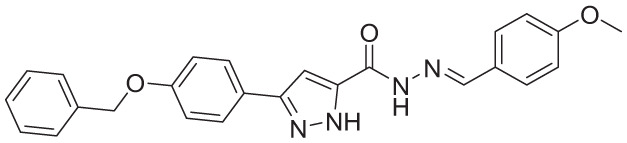 F1092-1608
7	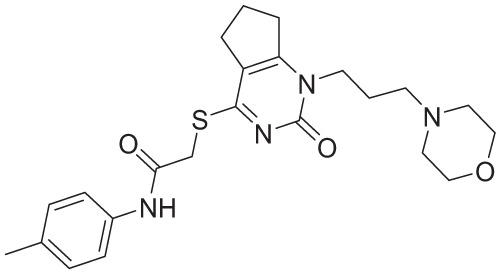 F2685-0114	8	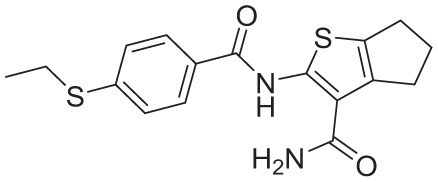 F2648-0093

### Structure-activity relationship analysis and quantitative structure-activity relationship modeling

The SAR heatmap generated for the 17 ATP synthase inhibitors revealed the presence of SubFP26, SubFP275, SubFP295, SubFP300, SubFP301, SubFP302, and SubFP307 predominantly in the highly active molecules. In contrast, SubFP2, SubFP3, SubFP173, and SubFP214 were mainly associated with low-activity compounds (Supplementary Table S3 and Figure S3). To identify structurally analogous molecules with potential inhibitory activity against Mtb ATP synthase, a Tanimoto similarity coefficient threshold of 40% was applied through chemical space mapping using a UMAP plot. This mapping aligned the 17 previously curated ATP synthase inhibitors with an antimycobacterial activity data set comprising 2168 molecules, based on substructure fingerprints. As a result, 1715 molecules exhibiting at least 40% structural similarity to one of the curated ATP synthase inhibitors were identified from the antimycobacterial assay data set.

A Venn diagram comparing the top 100 molecular features identified by VIP analysis for the 17 ATP synthase inhibitors and the 1715 anti-TB molecules revealed a 41% overlap in features. This substantial convergence in molecular characteristics between the 2 data sets further supported the selection of the 1715 experimentally tested anti-TB molecules as a hypothetical data set for QSAR modeling to elucidate and predict ATP synthase inhibition activity (Supplementary Figure S4).

Using the curated set of 1715 anti-TB molecules, QSAR modeling was initiated. Substructure fingerprints (n = 307) were calculated using the padelpy Python wrapper of the PaDEL-Descriptor software. Variance thresholding and Pearson correlation–based feature selection (correlation coefficient > 0.9) were applied to remove constant and highly correlated features. Following this feature elimination process, 114 molecular features were retained for QSAR model development. The data set was then split into training and test sets using an 80:20 ratio, resulting in 1434 molecules in the training set and 359 molecules in the test set. The SVM, ANN, RF, and XGBoost algorithms were subsequently implemented to develop individual ML-QSAR models.

Based on the initial modeling results, the ANN-based QSAR model demonstrated the best overall performance. The model achieved Pearson correlation coefficients of 0.9521 and 0.6702, RMSE values of 0.3231 and 0.8087, MSE values of 0.1044 and 0.6541, and MAE values of 0.1863 and 0.5485 for the training and test data sets, respectively. In addition, the model exhibited a 10-fold cross-validation score of 0.7761. To further improve model robustness, PCA was performed to detect potential outliers. The PCA identified 180 molecules from the combined training and test data sets that deviated from the main cluster and were classified as outliers. These molecules were removed prior to final model optimization.

Following outlier removal, the models were retrained using optimized hyperparameters through randomized search cross-validation. For the RF regressor, hyperparameter tuning included variations in the number of estimators (100, 200, 300, 500), maximum tree depth (None, 10, 20, 30), minimum samples required for splits (2, 5, 10), and feature selection methods (“sqrt,” “log2,” None). For the SVM regressor, kernel types (“rbf,” “poly,” “sigmoid”), regularization parameter C (0.1, 1, 10, 100), and gamma values (0.001, 0.01, 0.1, 1) were explored. Hyperparameter tuning for the XGBoost model included optimization of the number of estimators (100, 200, 300, 500), maximum tree depth (3, 5, 7, 10), learning rates (0.001, 0.01, 0.1, 0.2), and subsampling and feature selection ratios. For the ANN regressor, network architectures [(50), (100, 50), (100, 50, 25)], activation functions (“relu,” “tanh”), solver types (“adam,” “sgd”), learning rates (0.001, 0.01, 0.1), and iteration limits (500, 1000, 1500) were optimized.

**Figure 2.**
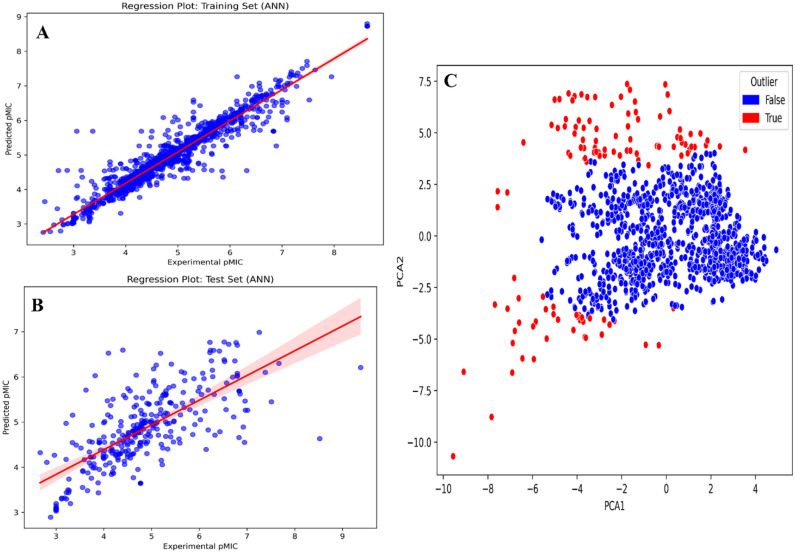
(A) and (B) Regression plots of training and test, respectively, of our final ANN-QSAR model and (C) outlier analysis during QSAR model development using PCA.

After hyperparameter optimization, the ANN model based on the outlier-removed substructure fingerprints continued to outperform other models. It achieved Pearson correlation coefficients of 0.9545 and 0.7067, RMSE values of 0.3309 and 0.7611, MSE values of 0.1095 and 0.5793, and MAE values of 0.2102 and 0.5183 for the training and test data sets, respectively. The optimized model also demonstrated a 10-fold cross-validation score of 0.7023. Overall, the outlier-removed substructure fingerprint–driven ANN-QSAR model exhibited the best accuracy and robustness among all trained models (Supplementary Table S4 and Figure 2).

### Feature elucidation of the machine learning-driven quantitative structure-activity relationship model for rational drug design

To further interpret the top 20 significant features of the ANN-QSAR model for the rational design of novel and more efficient inhibitors, VIP analysis, Pearson correlation analysis, and SHAP analysis were performed (Supplementary Table S5). In the substructure fingerprint–driven ANN-QSAR model, SubFP1, SubFP88, SubFP2, SubFP3, SubFP18, SubFP9, SubFP137, and SubFP181 exhibited the highest VIP scores, indicating their strong contribution to model performance.

To further investigate the nature of the relationships associated with these VIP-derived features, the Pearson correlation matrix and SHAP analyses were conducted. The Pearson correlation analysis identified SubFP9 (alkyl fluoride) as the most positively correlated feature with biological activity, highlighting the potential importance of fluorine atoms within alkyl groups. This feature may enhance bacterial cell penetration and facilitate weak dipolar interactions with Mtb ATP synthase. In addition, SubFP135 (vinylogous carbonyl or carboxyl derivative) showed a strong positive correlation with biological activity, suggesting its role in electronic delocalization and the presence of multiple hydrogen bond acceptor sites.

Other features demonstrating positive contributions to biological activity included SubFP137 (vinylogous ester), SubFP287 (conjugated double bond), and SubFP1 (primary carbon). In contrast, several features exhibited negative correlations with biological activity. Notably, SubFP3 (tertiary carbon) showed a negative correlation, indicating that bulky branching groups may hinder optimal molecular fitting within the ATP synthase active site. Additional negatively correlated features included SubFP5 (alkene), SubFP96 (carbodithioic ester), and SubFP88 (carboxylic acid derivative).

**Figure 3.**
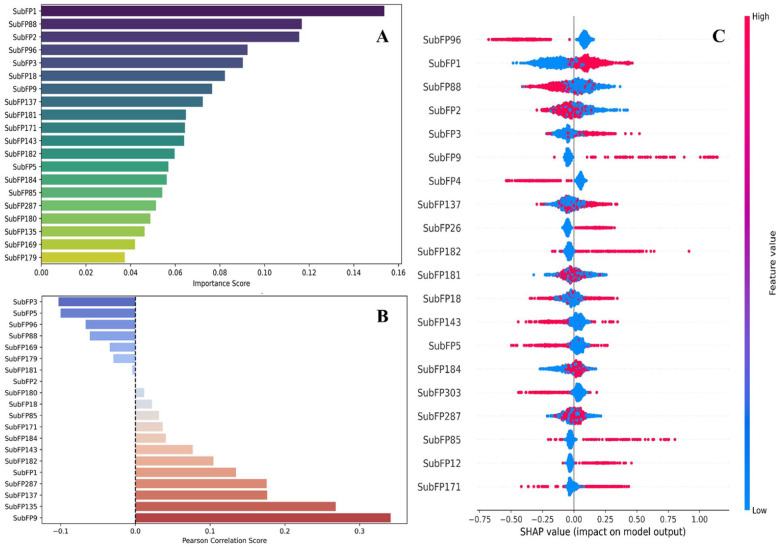
(A) VIP plot, (B) Pearson correlation plot, (C) SHAP analysis of the final ANN-QSAR model’s training data set to elucidate key molecular features contributing toward Mtb ATP synthase inhibition.

Overall, the analysis suggests that positively correlated features—such as alkyl fluorides, vinylogous systems, primary carbons, and conjugated ester moieties—may enhance molecular fitting, mimic enzymatic substrates, or strengthen non-covalent binding interactions. These properties likely contribute to improved biological activity through a combination of electronic, steric, and pharmacokinetic advantages (Figure 3A and B).

From the SHAP analysis, it was evident that features such as SubFP9 (alkyl fluoride), SubFP1 (primary carbon), SubFP88 (carboxylic acid derivative), and SubFP137 (vinylogous ester) exhibited strong positive SHAP values. In contrast, features including SubFP96 (carbodithioic ester), SubFP3 (tertiary carbon), SubFP4 (quaternary carbon), and SubFP5 (alkene) showed negative SHAP values.

To further support these observations, molecular feature–driven Tanimoto clustering analysis was performed on the high-activity and low-activity molecules within the QSAR data set. The analysis revealed that positively correlated and high-feature score molecular fingerprints—such as SubFP63, SubFP56, SubFP88, SubFP143, SubFP96, SubFP9, SubFP137, and SubFP181—identified through the Pearson correlation and SHAP analyses were overrepresented in clusters corresponding to highly active molecules. Conversely, molecular features including SubFP20, SubFP3, SubFP5, SubFP184, SubFP287, and SubFP171, which were negatively correlated with biological activity in both the SHAP and Pearson correlation analyses, were predominantly enriched in clusters associated with low-activity molecules within the QSAR data set (Figure 3C and Supplementary Figures S5 and S6).

### Prediction of the inhibition activity of the top 8 computational hits

The inhibition activity of Mtb ATP synthase was predicted using the ANN-QSAR model for the top 8 computational hits identified through the combined pharmacophore- and ADMET-driven virtual screening pipeline. Among these 8 hits, compounds F0526-1309 and F0526-1306 exhibited the highest predicted activity, with pMIC values of 5.295 and 5.628, respectively (Supplementary Table S6).

To further examine structural similarities among the top 8 hit molecules, a Tanimoto coefficient–based similarity analysis was performed, and shared molecular features were visualized using the WordCloud Python package. Several molecular features—such as SubFP1 (primary carbon), SubFP88 (carboxylic acid derivative), SubFP143 (carbonic acid derivative), SubFP9 (alkyl fluoride), SubFP137 (vinylogous ester), SubFP184 (heteroaromatic), SubFP26 (tertiary aliphatic amine), and SubFP171 (aryl chloride)—were consistently observed. These features had previously been identified as positive contributors to model predictions through SHAP and Pearson correlation analyses.

Notably, molecular features such as SubFP88, SubFP143, and SubFP13 were repeatedly highlighted in the WordCloud analysis of high-activity molecules (pMIC ≥ 8). In contrast, features including SubFP3 (tertiary carbon), SubFP5 (alkene), and SubFP96 (carbodithioic ester), which were associated with steric hindrance, were predominantly linked to low activity in both the SHAP and Pearson correlation analyses. Overall, the enrichment of high-activity–associated molecular features among the top 8 computational hits provides strong support for the predictive capability of the ANN-QSAR model and suggests that these molecules are structurally aligned with potent Mtb ATP synthase inhibitors (Supplementary Figure S7).

### Molecular docking study

Molecular docking serves as an integral tool for evaluating inhibitory activity, binding modes, and molecular interactions of the 8 hit candidates identified from pharmacophore- and QSAR-based screening. To validate the docking protocol, the co-crystallized ligand bedaquiline was first re-docked into the ATP synthase binding site (PDB ID: 8J57). The resulting pose showed near-complete alignment with the original co-crystallized ligand, confirming the robustness of the docking procedure, with a binding energy score of −5.394 kcal/mol (Supplementary Figure S8).

**Figure 4.**
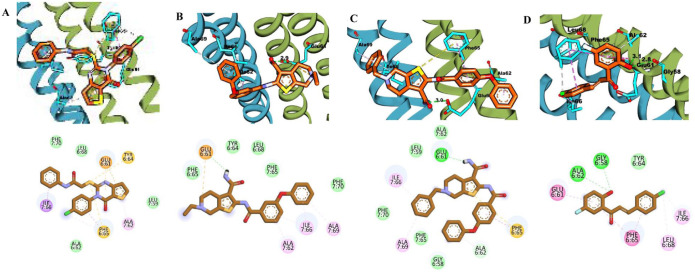
(A) 3D and 2D binding interactions of F0579-0616, (B) 3D and 2D binding interactions of F0526-1309, (C) 3D and 2D binding interactions of F0526-1306, and (D) 3D and 2D binding interactions of F1190-0509 within the ATP synthase binding pocket. In the 3D view, hits are shown in orange, amino acids in cyan, and the protein backbone in blue and green for chains 6 and 7, respectively.

Table 5 summarizes the docking scores and binding interactions of the 8 hit molecules alongside bedaquiline within the Mtb ATP synthase binding site. Based on the docking results, compounds F0579-0616, F0526-1309, F0526-1306, and F1190-0509 emerged as the most promising candidates, exhibiting binding scores ranging from −5.563 to −6.497 kcal/mol (Figure 4 and Supplementary Figure S9). The binding modes of these 4 top-ranked compounds closely resembled that of the reference ligand, bedaquiline.

**Table 5.** Docking scores (kcal/mol) besides binding interactions of the 8 hit candidates and bedaquiline as a reference ligand within the ATP synthase binding site.

**No**	Compound	Docking score (kcal/mol)	Binding interactions	
			H-bonds	Other interactions
1	F0579-0616	−5.686	-	Leu59, Glu61, Ala62, Tyr64, Phe65, Ile66, Leu68, Phe70.
2	F0526-1309	−5.715	Glu61	Glu61, Ala62, Tyr64, Phe65, Ile66, Leu68, Ala69, Phe70.
3	F0526-1306	−6.497	Glu61	Gly58, Leu59, Ala62, Phe65, Ile66, Phe70.
4	F1190-0509	−5.563	Gly58, Ala62.	Glu61, Phe65, Ile66, Leu68.
5	F1190-0598	−5.139	-	Glu61, Tyr64, Phe65, Ile66, Leu68, Ala69, Phe70.
6	F1092-1608	−5.495	Glu61	Gly58, Glu61, Ala62, Phe65, Ile66, Ala69, Phe70.
7	F2685-0114	−5.453	-	Gly58, Glu61, Ala62, Tyr64, Phe65, Ile66, Leu68.
8	F2648-0093	−4.998	Glu61	Glu61, Ala62, Tyr64, Phe65, Ile66,
9	BDQ	−5.394	Glu61	Gly58, Leu59, Glu61, Ala62, Tyr64, Phe65, Ile66, Leu68.

Compound F0579-0616 demonstrated a binding pattern comparable to bedaquiline, forming a π-anion interaction with Tyr64 and Phe65, hydrophobic interactions with Ala62 and Ile66, and van der Waals interactions with Ala62, Leu68, and Phe70. Similarly, compounds F0526-1309 and F0526-1306 engaged in key binding interactions, particularly through hydrogen bonding with Glu61. Compound F0526-1309 additionally exhibited an attractive charge interaction with Glu61, hydrophobic interactions with Ala62, Ile66, and Ala69, and van der Waals interactions with Tyr64, Phe65, Leu68, and Phe70. In contrast, compound F0526-1306 formed a π-sulfur interaction with Phe65, hydrophobic interactions with Ile66 and Ala69, and van der Waals interactions with Gly58, Leu59, Ala62, Phe65, and Phe70.

Finally, compound F1190-0509 formed 2 hydrogen bonds with Gly58 and Ala62, along with hydrophobic interactions involving Glu61, Phe65, Ile66, and Leu68, and van der Waals interactions with Tyr64.

### Molecular dynamics simulations and binding free energy analyses

**Figure 5.**
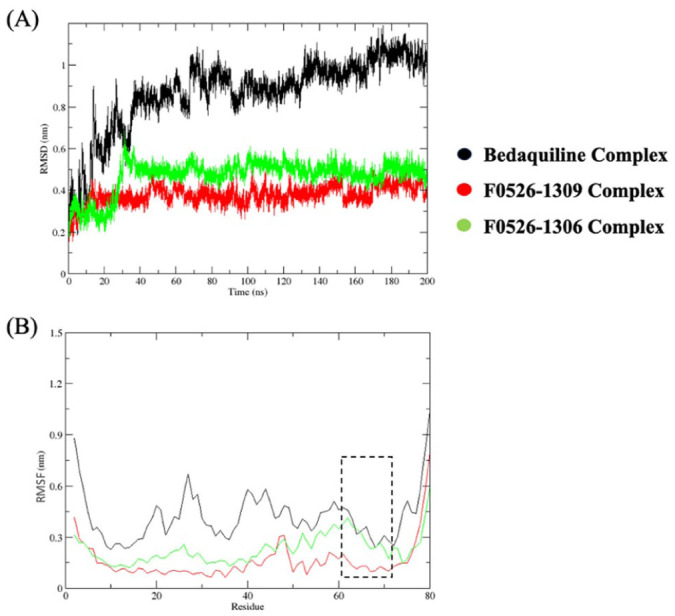
The root mean square deviation (RMSD) and the root mean square fluctuation (RMSF) of the ATP synthase complexes with bedaquiline (black lines), F0526-1309 (red lines), and F0526-1306 (green lines) compounds observed during the MD simulations performed for a period of 200 ns are shown in panels (A) and (B), respectively.

**Figure 6.**
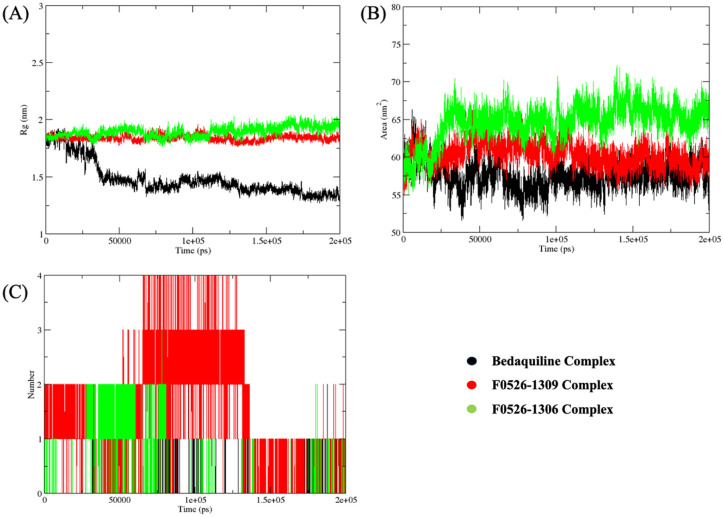
Post-molecular dynamics (MD) analyses of ATP synthase complexes with bedaquiline (black lines), F0526-1309 (red lines), and F0526-1306 (green lines). Radius of gyration (Rg), solvent-accessible surface area (SASA), and the number of intermolecular hydrogen bonds formed in the complexes over a period of 200 ns are shown in panels (A), (B), and (C), respectively.

To characterize the dynamic interactions of protein-ligand complexes within the body, the top 2 hits from the predictions from the QSAR and docking study were further subjected to MD simulations. Hence, the F0526-1309 and F0526-1306 complexes with Mtb ATP synthase were assessed in comparison to the reference bedaquiline molecule. The analysis of the generated ligand-protein trajectories indicated that the 2 hit candidates displayed enhanced stability and a more rigid dynamic profile compared to the co-crystallized reference, bedaquiline (Figures 5 and 6). The RMSD analysis revealed that both lead compounds formed highly stable complexes with ATP synthase throughout the 200 ns simulation. The F0526-1309 complex exhibited the lowest average RMSD (0.39 nm), followed by F0526-1306 (0.49 nm), compared to bedaquiline (0.95 nm). This indicates reduced conformational drift and enhanced structural stability for the novel inhibitors relative to the reference drug. Residue-wise RMSF analysis demonstrated suppressed fluctuations across key binding site residues for F0526-1309 and F0526-1306 complexes, suggesting tight ligand engagement and reduced local flexibility displaying the lowest atomic fluctuations, with a value of 0.16 nm and 0.27 nm. In contrast, the bedaquiline-bound complex showed comparatively higher fluctuations (0.42 nm), reflecting greater structural adaptability.

The Rg analysis indicated that both F0526-1309 (1.84 nm) and F0526-1306 (1.90 nm) induced slightly more expanded yet stable Mtb ATP synthase conformations compared to bedaquiline (1.41 nm). This controlled expansion reflects adaptive accommodation of the ligands without compromising global structural integrity. Solvent-accessible surface area analysis also falls in agreement with these observations. The lead compounds displayed marginally higher SASA values (60.28 nm^2^ for F0526-1309 and 65.34 nm^2^ for F0526-1306) compared to bedaquiline (57.33 nm^2^), suggesting enhanced solvent exposure consistent with stable but dynamically accessible binding conformations. Hydrogen bond analysis showed that the complexes with F0526-1309 and F0526-1306 compounds are more stable than that of bedaquiline. The observed increase in hydrogen bonding frequency for F0526-1309 contributes to its superior structural stabilization within the ATP synthase binding pocket.

The Molecular Mechanics Poisson-Boltzmann Surface Area (MMPBSA) calculations revealed favorable binding free energies for all 3 complexes. Bedaquiline exhibited the most negative ΔGTOTAL (−26.33 kcal/mol), closely followed by F0526-1306 (−25.86 kcal/mol), while F0526-1309 showed a ΔGTOTAL of −21.77 kcal/mol (Table 6). The energy decomposition analysis highlighted that the complex formation was primarily driven by strong van der Waals and electrostatic interactions. Notably, both lead compounds exhibited significantly enhanced electrostatic contributions compared to bedaquiline, which were partially offset by higher polar solvation penalties. Such significant contributions of energy components explain their enhanced binding affinities.

**Table 6.** Binding free energy of bedaquiline, F0526-1309, and F0526-1306 with ATP synthase.

Serial number	Components of binding free energy (kcal/mol)							
	Complexes	ΔVDWAALS (van der Waals energy)	ΔEEL (Electrostatic energy)	ΔEPOLAR (Polar solvation energy)	ΔENPOLAR (Non-polar solvation energy)	ΔGGAS (Gas-phase free energy)	ΔGSOLV (Solvation free energy)	ΔGTOTAL (Total binding free energy)
1	Bedaquiline	−32.15	−35.29	44.61	−3.50	−67.44	41.12	−26.33
2	F0526-1309	−24.43	−147.15	152.25	−2.44	−171.58	149.81	−21.77
3	F0526-1306	−28.63	−112.27	117.48	−2.44	−140.90	115.04	−25.86

### Validation of the computational hits using in vitro inhibition of *Mycobacterium marinum*

**Figure 7.**
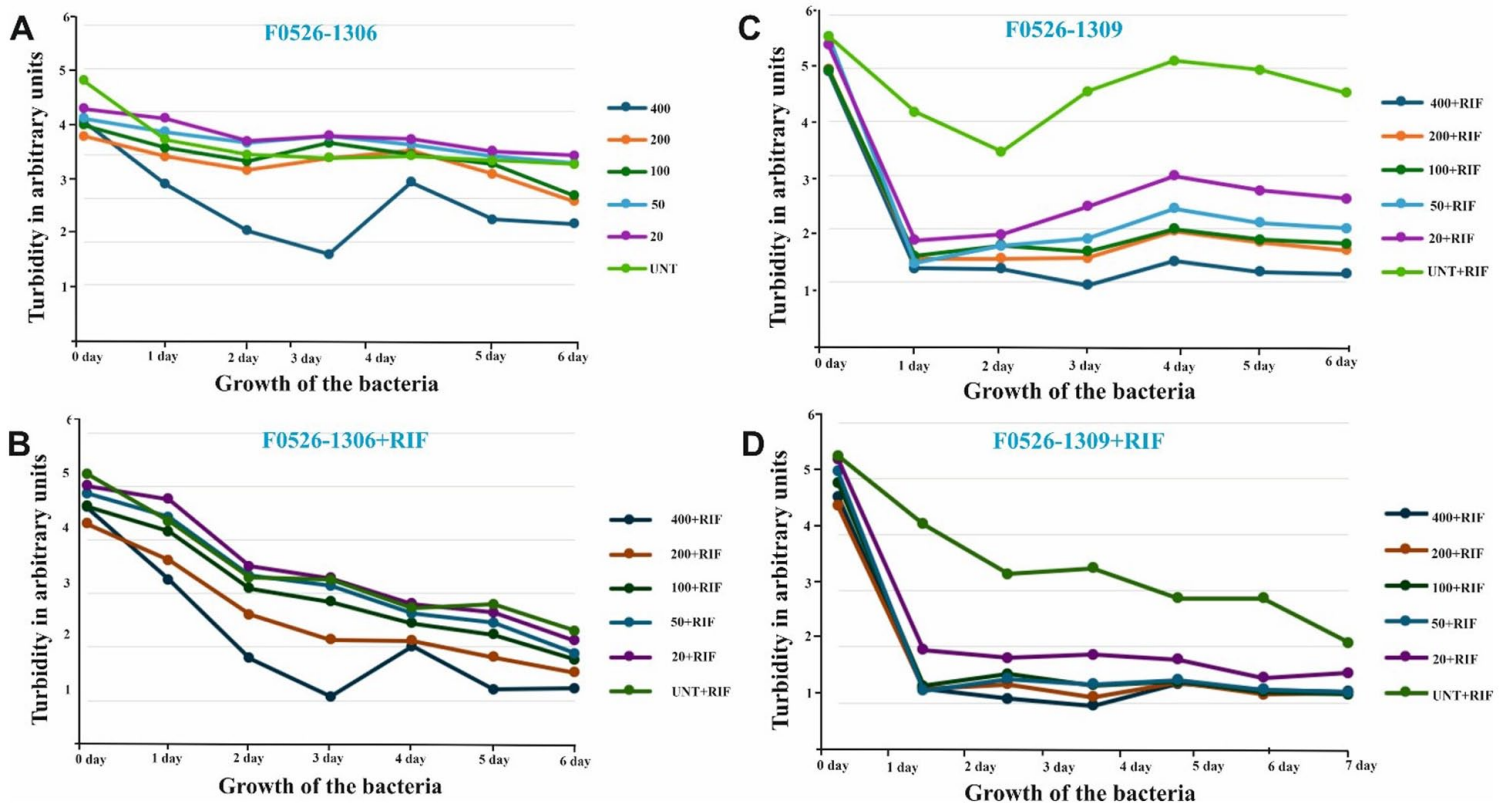
Inhibition of *Mycobacterium marinum* growth under different treatment conditions. (A) Treatment with compound F0526-1306 alone resulted in a reduction in bacterial growth. (B) No significant inhibition was observed when F0526-1306 was combined with rifampicin. (C) Treatment with compound F0526-1309 alone showed a dose-dependent inhibition of bacterial growth. (D) F0526-1309, in combination with rifampicin, also inhibited bacterial growth, indicating potential synergistic activity. (UNT, untreated control; RIF, rifampicin alone at a fixed concentration of 400 µg/mL; Compound, test compounds at 20-400 µM; Compound + RIF, combination treatment).

To evaluate whether the computationally identified hit molecules inhibit the growth of *M marinum* in vitro, we conducted standard inhibition assays using liquid cultures in 96-well microtiter plates. These experiments were designed to validate our computational findings. In addition to visual inspection, bacterial growth was monitored by measuring the optical density (OD) over a 0- to 7-day period. In preliminary experiments, we tested a concentration range of 25 µM to 400 µM. Treatment with the molecule F0526-1306 at 400 µM resulted in a noticeable reduction in bacterial growth 3 days post-treatment. However, when F0526-1306 was administered in combination with rifampicin, no significant reduction in bacterial growth was observed. A clear dose-dependent inhibition of bacterial growth was observed by F0526-1306 alone. Similarly, treatment with molecule F0526-1309 led to a dose-dependent reduction in bacterial growth. Notably, F0526-1309 inhibited bacterial growth both as a monotherapy and in combination with rifampicin. These results suggest that F0526-1309, in particular, may act synergistically with rifampicin (Figure 7).

### Toxicity studies of the computational hits using zebrafish models

**Figure 8.**
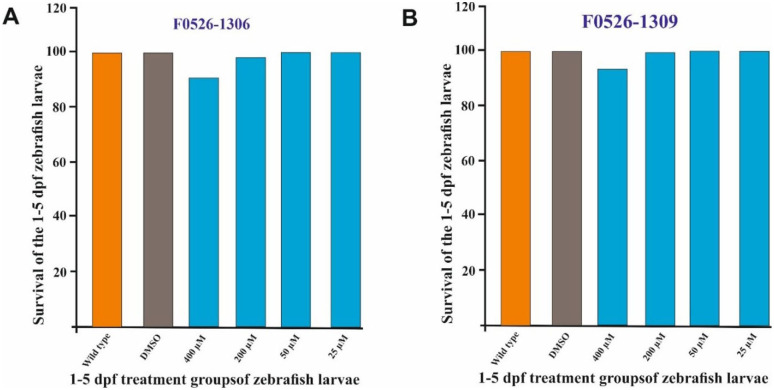
(A) Survival of zebrafish larvae 5 days post-treatment with compound F0526-1306. (B) A bar graph depicting the survival of 5-day post-fertilization (dpf) zebrafish larvae following treatment with compound F0526-1309.

The safety of both compounds following a 5-day exposure in developing zebrafish embryos is shown in Figure 8. Both molecules were found to be non-toxic even at the highest tested concentration of 400 µM. These results indicate that the molecules are safe for further in vivo studies, consistent with findings from our previous research involving other molecules.^29,37^ To assess the toxic effects of the inhibitors on developing zebrafish larvae, we analyzed 8 observable phenotypic parameters: hatching, heartbeat, otolith sac development, yolk sac utilization, movement pattern, body shape, swim bladder development, and edema. These parameters were monitored using a stereomicroscope, and observations were recorded for each treatment group and compared to control embryos, which were either untreated or treated with 1% DMSO. None of the molecules tested induced any defects in the parameters studied. Toxicity assessment showed that not only was the survival of the zebrafish larvae good, but the molecules also did not induce any observable phenotypic defects, and the phenotypes were comparable with the control group embryos (wild type not treated with any molecule) and the DMSO-treated group.

### Prediction of molecular mechanisms of final hit molecules at the host-pathogen interface

We further investigated the immunomodulatory mechanisms of action of the 2 best-performing molecules, F0526-1306 and F0526-1309, to better understand their potential interactions within the host during tuberculosis infection. For compound F0526-1309, a total of 100 unique target genes were identified using the SwissTargetPrediction server. Protein-protein interaction data for these targets were subsequently curated from the STRING database, and network analysis using the degree method implemented in the cytoHubba plugin of Cytoscape identified GSK3B, MTOR, EGFR, SRC, and PIK3CA as the top 5 hub genes.

**Figure 9.**
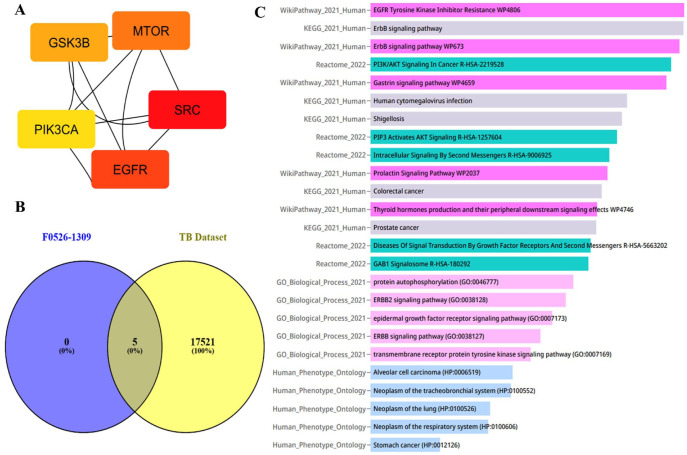
Prediction of anti-TB molecular mechanism of molecule F0526-1309 at host pathogen interface: (A) top 5 hub genes using degree method, (B) Venn plot analysis of top 5 hub genes against CTD database TB gene data set, and (C) pathway enrichment and gene ontology analysis of the top 5 hub genes using Enrichr-KG platform.

The Venn diagram analysis comparing these hub genes with a large tuberculosis-associated gene data set from the CTD confirmed their strong association with TB-related disease mechanisms. Gene Ontology and pathway enrichment analyses were then performed using the Enrichr-KG webserver (Figure 9). The most significantly enriched pathways included EGFR tyrosine kinase inhibitor resistance, ErbB signaling pathway, ERBB2 signaling pathway, epidermal growth factor receptor signaling, PIP3-mediated AKT signaling, PI3K/AKT signaling in cancer, intracellular signaling by second messengers, diseases of signal transduction by growth factor receptors, protein autophosphorylation, and transmembrane receptor protein tyrosine kinase signaling.

For the molecule F0526-1306, a total of 100 unique target genes were identified using the SwissTargetPrediction server. Protein-protein interaction data for the predicted targets of F0526-1306 were then curated from the STRING database, and network analysis using the degree method implemented in the cytoHubba plugin of Cytoscape identified CCND1, MTOR, EGFR, ERBB2, and PIK3CA as the top 5 hub genes. The Venn diagram analysis comparing these hub genes with a large tuberculosis-associated gene data set from the Comparative Toxicogenomics Database (CTD) confirmed their strong association with TB-related disease mechanisms.

The enrichment of EGFR-ERBB2 family–associated pathways suggests that F0526-1309 may contribute to the restoration of epithelial signaling, enhancement of host immune recognition, and disruption of the intracellular niche exploited by Mtb. Furthermore, enrichment of pathways associated with PI3K/AKT/mTOR signaling, second messengers, and signal transduction indicates that F0526-1309 may have the potential to reactivate autophagy, reset dysregulated signaling cascades, and promote host-mediated clearance of intracellular mycobacteria.

Single-cell RNA expression analysis revealed that the hub genes MTOR, GSK3B, and PIK3CA were highly expressed in macrophages, whereas SRC and EGFR showed predominant expression in both epithelial cells and macrophages (Supplementary Figures S10 to S12). Collectively, these systems biology analyses suggest that F0526-1309 primarily exerts its host-directed effects through macrophages, thereby targeting key intracellular survival mechanisms utilized by Mtb.

**Figure 10.**
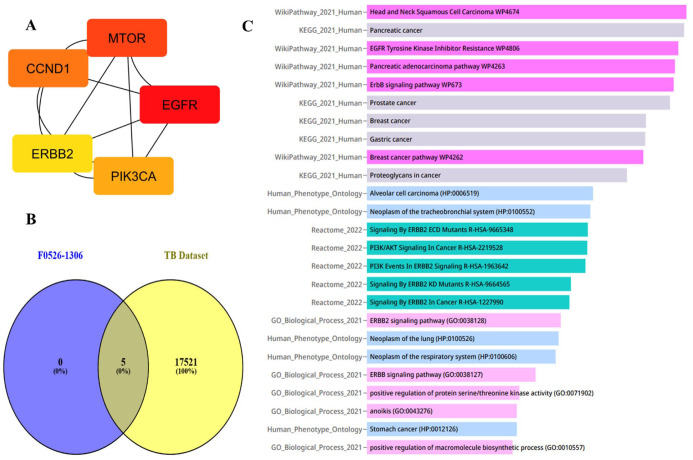
Prediction of anti-TB molecular mechanism of molecule F0526-1306 at host pathogen interface: (A) top 5 hub genes using degree method, (B) Venn plot analysis of top 5 hub genes against CTD database TB gene data set, and (C) pathway enrichment and gene ontology analysis of the top 5 hub genes using Enrichr-KG platform.

Gene Ontology and pathway enrichment analyses were subsequently performed using the Enrichr-KG webserver (Figure 10). The most significantly enriched pathways included ERBB2 signaling, signaling by ERBB2 ECD/KD mutants, PI3K events in ERBB2 signaling, positive regulation of protein serine/threonine kinase activity, anoikis, and positive regulation of macromolecule biosynthetic processes. Enrichment of ERBB2-centric signaling pathways suggests that F0526-1306 may contribute to restoring immune surveillance and reducing cellular environments favorable to bacterial persistence. In addition, enrichment of PI3K-associated pathways and kinase-regulated survival signaling indicates a potential mechanism through which F0526-1306 may promote autophagic clearance and enhance immune-mediated elimination of intracellular pathogens.

Single-cell RNA expression analysis revealed that hub genes ERBB2 and CCND1 were highly expressed in epithelial cell populations, including club cells, endothelial cells, and fibroblasts. The CCND1 also showed elevated expression in T2P epithelial-like cells. Furthermore, hub genes EGFR and PIK3CA exhibited high expression across multiple epithelial and immune cell populations (Supplementary Figures S10 to S12). Collectively, this systems biology analysis suggests that F0526-1306 primarily exerts its host-directed effects through epithelial dynamics, potentially disrupting the cellular niche and extracellular matrix components that support Mtb persistence during chronic infection.

### Limitations

Although the integrated computational pipeline employed in this study identified compounds with favorable target-level binding, stability, and predicted activity, it is important to acknowledge that whole-cell antimycobacterial inhibition is influenced by additional biological factors that are not fully captured by silico models. These include mycobacterial cell wall permeability, compound uptake and efflux mechanisms, metabolic adaptation, and growth-state–dependent sensitivity. Consequently, strong predicted target engagement does not always translate into pronounced growth inhibition in vitro.

In addition, while the present study focused on structure-based discovery and preliminary biological validation, direct enzyme inhibition assays using purified ATP synthase were not performed. Such assays would provide definitive mechanistic confirmation of ATP synthase inhibition and will be pursued in future studies to further advance the identified hit compounds toward lead optimization.

## Conclusion

Given the increasing drug resistance of Mtb, this study employed a multidimensional computational strategy to identify and design novel, selective small-molecule inhibitors targeting ATP synthase, a central regulator of mycobacterial energy metabolism. A receptor-based pharmacophore modeling approach was first implemented using LigandScout, based on the crystal structures of ATP synthase complexes (PDB IDs: 8J57, 8JR1, and 8G0B). Among the 10 generated pharmacophore models, Ph4-7 emerged as the best-performing model, comprising 2 hydrophobic features, 1 hydrogen bond donor feature, and 7 exclusion volumes. This model successfully identified 78 hits, including 14 TPs, achieving a sensitivity of 0.70 and a specificity of 0.936. Furthermore, Ph4-7 demonstrated strong predictive performance, with a yield of 0.179, an enrichment factor of 6.372, and an AUC value of 0.81, indicating moderate-to-excellent classification capability.

To further refine candidate selection, ML-QSAR modeling and ADMET-based filtering were applied, resulting in the identification of 8 promising hit molecules. Subsequent molecular docking using AutoDock Vina, combined with QSAR-based predictions, highlighted 2 lead candidates, F0526-1309 and F0526-1306. Both molecules exhibited favorable binding interactions within the ATP synthase active site, closely resembling those of the reference inhibitor bedaquiline. Notably, F0526-1306 demonstrated strong and stable interactions, including a π-sulfur interaction with Phe65, hydrogen bonding with Glu61, hydrophobic contacts with Ile66 and Ala69, and additional van der Waals interactions with Gly58, Leu59, Ala62, Phe65, and Phe70, collectively supporting its stable accommodation within the binding pocket.

Finally, MD simulations using GROMACS were performed on the 2 most promising compounds, F0526-1306 and F0526-1309. The complex with F0526-1309 exhibited stable dynamics with the least variation around the RMSD value of 0.39 nm, when compared to the complex with F0526-1306 (0.49 nm). The stable dynamics of F0526-1309 complex is also ensured by the observed confined atomic fluctuations (RMSF) falling closer to 0.16 nm than the complex with F0526-1306 compound (0.27 nm). Hydrogen bond analyses on both complexes also supported the stable complex formation of F0526-1309 complex than that of F0526-1306. In addition, the binding free energy analyses revealed the significant contribution of both van der Waals and electrostatic interactions in stabilizing the F0526-1309 and F0526-1306 complexes when compared with bedaquiline.

Experimental validation through antimycobacterial inhibition assays demonstrated that both F0526-1306 and F0526-1309 significantly reduced mycobacterial biofilm formation. Importantly, toxicity assessments conducted using a zebrafish model confirmed that both molecules were safe and well tolerated, supporting their suitability for further in vivo investigation.

In summary, the integrative computational-experimental framework employed in this study successfully identified 2 promising ATP synthase inhibitors, F0526-1306 and F0526-1309, which exhibit strong binding interactions, favorable dynamic stability, and acceptable safety profiles. These findings provide a solid foundation for further optimization and experimental validation, underscoring the potential of these molecules as future antitubercular drug candidates.

## Supplemental Material

sj-docx-1-bbi-10.1177_11779322261438313 – Supplemental material for Combating Drug Resistance in Mycobacterium Tuberculosis: A Combinatorial in Silico and Experimental Modeling Approach Toward Novel ATP Synthase Inhibitor Discovery Supplemental material, sj-docx-1-bbi-10.1177_11779322261438313 for Combating Drug Resistance in Mycobacterium Tuberculosis: A Combinatorial in Silico and Experimental Modeling Approach Toward Novel ATP Synthase Inhibitor Discovery by Haidy H. El-Zoheiry, Ratul Bhowmik, Ajay Manaithiya, Rajarshi Ray, Mahesh Samantaray, Amutha Ramaswamy and Ashok Aspatwar in Bioinformatics and Biology Insights

sj-xlsx-2-bbi-10.1177_11779322261438313 – Supplemental material for Combating Drug Resistance in Mycobacterium Tuberculosis: A Combinatorial in Silico and Experimental Modeling Approach Toward Novel ATP Synthase Inhibitor Discovery Supplemental material, sj-xlsx-2-bbi-10.1177_11779322261438313 for Combating Drug Resistance in Mycobacterium Tuberculosis: A Combinatorial in Silico and Experimental Modeling Approach Toward Novel ATP Synthase Inhibitor Discovery by Haidy H. El-Zoheiry, Ratul Bhowmik, Ajay Manaithiya, Rajarshi Ray, Mahesh Samantaray, Amutha Ramaswamy and Ashok Aspatwar in Bioinformatics and Biology Insights
