# Supplementary material for: Combating Drug Resistance in Mycobacterium Tuberculosis: A Combinatorial in Silico and Experimental Modeling Approach Toward Novel ATP Synthase Inhibitor Discovery
Source: Bioinform Biol Insights. 2026 Apr 8;20:11779322261438313. doi: 10.1177/11779322261438313 (PMC13065274; doi:10.1177/11779322261438313)
Supplement: sj-docx-1-bbi-10.1177_11779322261438313 – Supplemental material for Combating Drug Resistance in Mycobacterium Tuberculosis: A Combinatorial in Silico and Experimental Modeling Approach Toward Novel ATP Synthase Inhibitor Discovery [file sj-docx-1-bbi-10.1177_11779322261438313.docx]

**Combating Drug Resistance in Mycobacterium tuberculosis: A combinatorial In Silico and Experimental Approach towards novel ATP Synthase Inhibitor Discovery**

Haidy Hany AbdElHamid ElZohiery^1#^, Ratul Bhowmik^2#^, Ajay Manaithiya^2#^, Rajarshi Ray^2^, Ashok Aspatwar^2*^

^1^Department of Pharmaceutical Chemistry, Cairo University, Egypt

^2^Faculty of Medicine and Health Technology, Tampere University, Tampere, 33100, Finland

# All the authors contributed equally

**Corresponding author:** Dr. Ashok Aspatwar ([ashok.aspatwar@tuni.fi](mailto:ashok.aspatwar@tuni.fi))

**Table S1**: The collected F-ATP synthase inhibitors used for pharmacophore selection and validation in test set.

| **#** | **Structure** | **Ref.** | **#** | **Structure** | **Ref.** |
| --- | --- | --- | --- | --- | --- |
| **1** |  | [1] | **2** |  | [2] |
| **3** |  | [2] | **4** |  | [2] |
| **5** |  | [2] | **6** |  | [3] |
| **7** |  | [3] | **8** |  | [8] |
| **9** |  | [4] | **10** |  | [4] |
| **11** |  | [4] | **12** |  | [4] |
| **13** |  | [5] | **14** |  | [5] |
| **15** |  | [5] | **16** |  | [6] |
| **17** |  | [6] | **18** |  | [7] |
| **19** |  | [7] | **20** |  | [9] |

***Table S2****: Modalities for calculating performance parameters.*

| **#** | **Parameter** | **Equation** | **Justification** |
| --- | --- | --- | --- |
| **1** | **Selectivity (Se)** | $Se=\frac{TP}{A}$ | Accuracy in choosing active compounds. |
| **2** | **Specificity (Sp)** | $Sp=\frac{TN}{N-A}$ | Discriminate against decoys. |
| **3** | **Yield of actives (Ya)** | $Ya=\frac{TP}{n}$ | Conveys the hit rate. |
| **4** | **Enrichment (E)** | $E=\frac{Tp/n}{A/N}$ | Evaluates the success rate of virtual screening compared to random selection methods in identifying active compounds. |
| **5** | **Accuracy (acc)** | $acc=\frac{Tp+TN}{N}$ | Differentiate between  actives and decoys in virtual screening workflow. |
| **6** | **Discrimination ratio (DR)** | $DR=\frac{Se}{Sp}$ | Predicts accuracy in distinguishing actives and decoys in the virtual screening process. |
| **7** | **F1 score (F1)** | $F1=\frac{TP}{TP+\frac{1}{2} (FP+FN)}$ | Appraise the overall quality of the pharmacophore  model in discriminating between actives and  decoys (0 - 1). |
| **8** | **Mathew’s Correlation Coefficient (MCC)** | $MCC=\frac{\left( TPxTN \right)-(FPxFN)}{\sqrt{(TP+FN)(TP+FP)(TN+FP)(TN+FN)}}$ | Generates a high score when the pharmacophore correctly predicts a high number of actives and decoys. |

**N** = the total number of compounds in the dataset, **TP** = true positive, **TN** = true negative, **A** = the number of actives, **n** = hits count, **FP** = false positive, **FN** = false negative.

***Table S3:*** *Key molecular features from SAR analysis of 17 previously discovered Mtb ATP synthase inhibitors*

| Fingerprints | Interpretation |
| --- | --- |
| SubFP1 | Primary carbon |
| SubFP2 | Secondary carbon |
| SubFP3 | Tertiary carbon |
| SubFP12 | Alcohol |
| SubFP15 | Tertiary alcohol |
| SubFP18 | Alkylarylether |
| SubFP26 | Tertiary aliph amine |
| SubFP169 | Phenol |
| SubFP171 | Arylchloride |
| SubFP173 | Arylbromide |
| SubFP181 | Hetero N nonbasic |
| SubFP184 | Heteroaromatic |
| SubFP214 | Sulfonic derivative |
| SubFP274 | Aromatic |
| SubFP275 | Heterocyclic |
| SubFP287 | Conjugated double bond |
| SubFP295 | C ONS bond |
| SubFP300 | 1,3-Tautomerizable |
| SubFP301 | 1,5-Tautomerizable |
| SubFP302 | Rotatable bond |
| SubFP307 | Chiral center specified |

***Table S4:*** *Detailed statistics of all the developed ML-driven QSAR models*

| **Algorithm** | **Train RMSE** | **Test RMSE** | **CV RMSE** | **Train MSE** | **Test MSE** | **Train MAE** | **Test MAE** | **Train Pearson** | **Test Pearson** |
| --- | --- | --- | --- | --- | --- | --- | --- | --- | --- |
|  |  |  |  |  |  |  |  |  |  |
| **Initial Model** |  |  |  |  |  |  |  |  |  |
| **Random Forest** | 0,3590 | 0,7569 | 0,6840 | 0,1289 | 0,5729 | 0,2328 | 0,4907 | 0,9436 | 0,7072 |
| **SVM** | 0,5987 | 0,7911 | 0,7161 | 0,3584 | 0,6258 | 0,3992 | 0,5466 | 0,8306 | 0,6775 |
| **XGBoost** | 0,4036 | 0,7574 | 0,7001 | 0,1629 | 0,5736 | 0,2589 | 0,5002 | 0,9253 | 0,7071 |
| **ANN** | 0,3231 | 0,8087 | 0,7761 | 0,1044 | 0,6541 | 0,1863 | 0,5485 | 0,9521 | 0,6702 |
|  |  |  |  |  |  |  |  |  |  |
| **Outliers removed** |  |  |  |  |  |  |  |  |  |
| **Random Forest** | 0,3554 | 0,7787 | 0,6961 | 0,1263 | 0,6064 | 0,2283 | 0,5049 | 0,9452 | 0,6916 |
| **SVM** | 0,5938 | 0,8018 | 0,7179 | 0,3526 | 0,6428 | 0,3923 | 0,5426 | 0,8360 | 0,6806 |
| **XGBoost** | 0,3951 | 0,7965 | 0,6938 | 0,1561 | 0,6345 | 0,2446 | 0,5268 | 0,9294 | 0,6766 |
| **ANN** | 0,3230 | 0,8520 | 0,7870 | 0,1043 | 0,7258 | 0,1888 | 0,5922 | 0,9541 | 0,6330 |
|  |  |  |  |  |  |  |  |  |  |
| **Outliers removed and hypertuned parametrization** |  |  |  |  |  |  |  |  |  |
| **Random Forest** | 0,3629 | 0,7619 | 0,6785 | 0,1317 | 0,5805 | 0,2369 | 0,4962 | 0,9446 | 0,7122 |
| **SVM** | 0,7164 | 0,8559 | 0,7729 | 0,5132 | 0,7326 | 0,4971 | 0,6087 | 0,7454 | 0,6325 |
| **ANN (Best Model)** | **0,3309** | **0,7611** | **0,7023** | **0,1095** | **0,5793** | **0,2102** | **0,5183** | **0,9545** | **0,7067** |
| **XGBoost** | 0,3951 | 0,7965 | 0,6938 | 0,1561 | 0,6345 | 0,2446 | 0,5268 | 0,9294 | 0,6766 |

***Table S5:*** *Top molecular features from VIP plot, Pearson correlation plot, and SHAP analysis of the final ANN-QSAR model*

| **VIP Plot analysis identified Fingerprints** | **Interpretation** | **SHAP analysis identified Fingerprints** | **Interpretation** |
| --- | --- | --- | --- |
| SubFP1 | Primary carbon | SubFP96 | Carbodithioic ester |
| SubFP88 | Carboxylic acid derivative | SubFP1 | Primary carbon |
| SubFP2 | Secondary carbon | SubFP88 | Carboxylic acid derivative |
| SubFP96 | Carbodithioic ester | SubFP2 | Secondary carbon |
| SubFP3 | Tertiary carbon | SubFP3 | Tertiary carbon |
| SubFP18 | Alkylarylether | SubFP9 | Alkylfluoride |
| SubFP9 | Alkylfluoride | SubFP4 | Quaternary carbon |
| SubFP137 | Vinylogous ester | SubFP137 | Vinylogous ester |
| SubFP181 | Hetero N nonbasic | SubFP26 | Tertiary aliph amine |
| SubFP171 | Arylchloride | SubFP182 | Hetero O |
| SubFP143 | Carbonic acid derivatives | SubFP181 | Hetero N nonbasic |
| SubFP182 | Hetero O | SubFP18 | Alkylarylether |
| SubFP5 | Alkene | SubFP143 | Carbonic acid derivatives |
| SubFP184 | Heteroaromatic | SubFP5 | Alkene |
| SubFP85 | Carboxylic ester | SubFP184 | Heteroaromatic |
| SubFP287 | Conjugated double bond | SubFP303 | Michael acceptor |
| SubFP180 | Hetero N basic no H | SubFP287 | Conjugated double bond |
| SubFP135 | Vinylogous carbonyl or carboxyl derivative | SubFP85 | Carboxylic ester |
| SubFP169 | Phenol | SubFP12 | Alcohol |
| SubFP179 | Hetero N basic H | SubFP171 | Arylchloride |

***Table S6:*** *ANN-QSAR model predictions for the top 8 pharmacophore modeling hits*

| Name | Predicted pMIC |
| --- | --- |
| F0579-0616 | 4.249 |
| **F0526-1309** | **5.295** |
| **F0526-1306** | **5.628** |
| F1190-0509 | 4.291 |
| F1190-0598 | 4.731 |
| F1092-1608 | 3.692 |
| F2685-0114 | 4.106 |
| F2648-0093 | 4.952 |

***
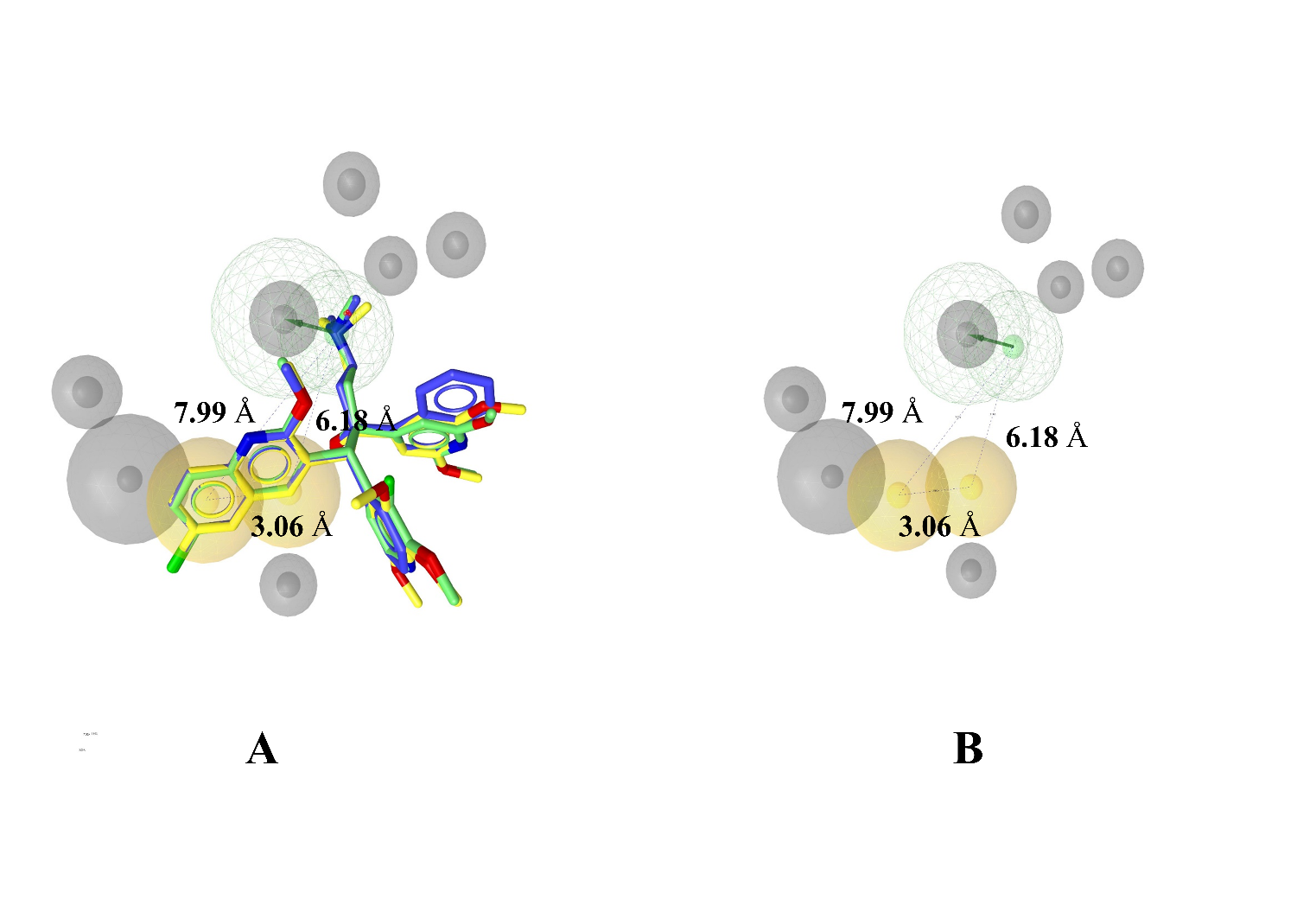
***

***Figure S1****: The opted pharmacophoric model, "Ph4-7”, highlighting key features and the distances between them. Bedaquiline is depicted in blue, TBAJ-587 in green, and TBAJ-876 in yellow.*

*
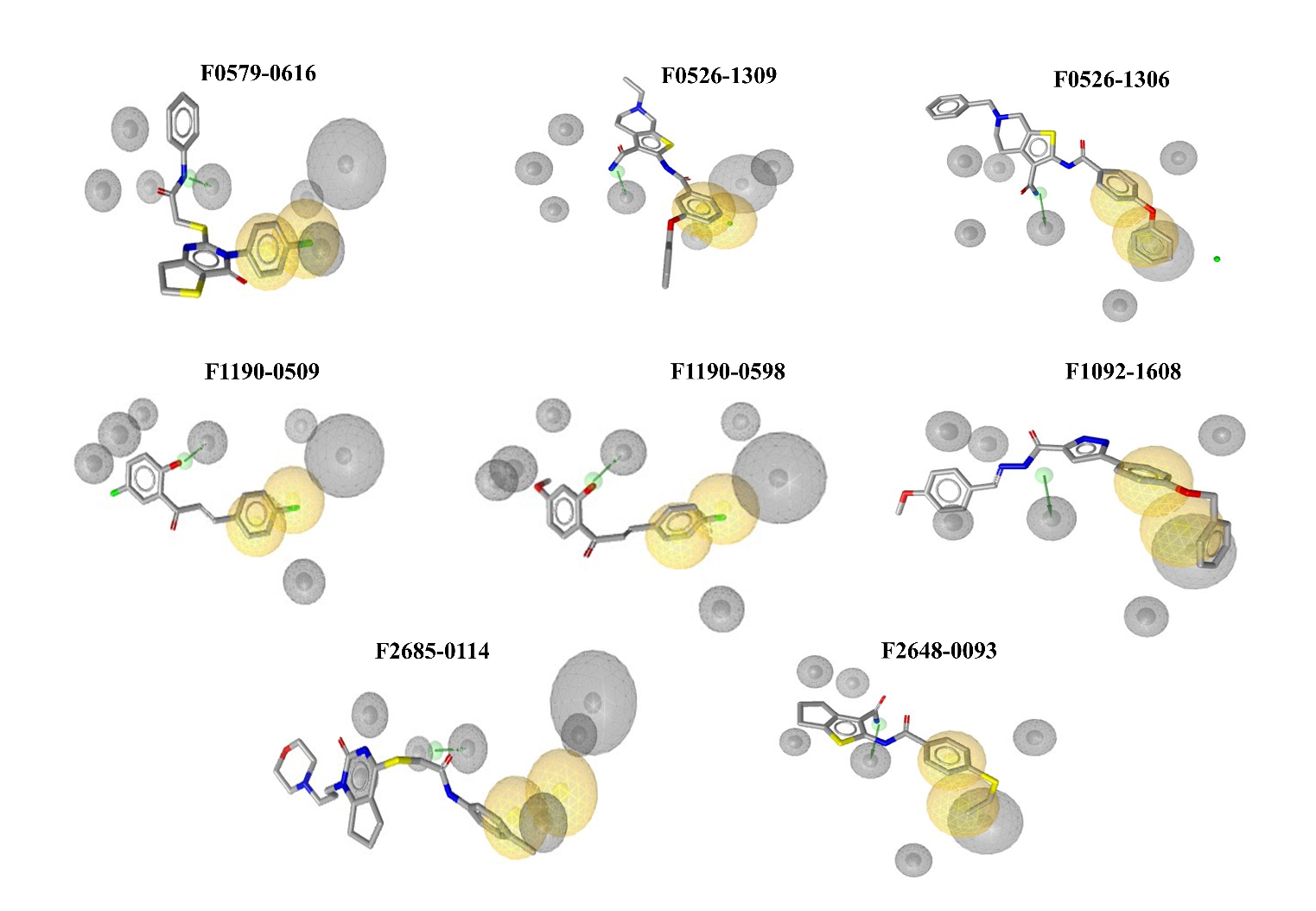
*

***Figure S2****: Fit of the hit compounds to the pharmacophore model*

*
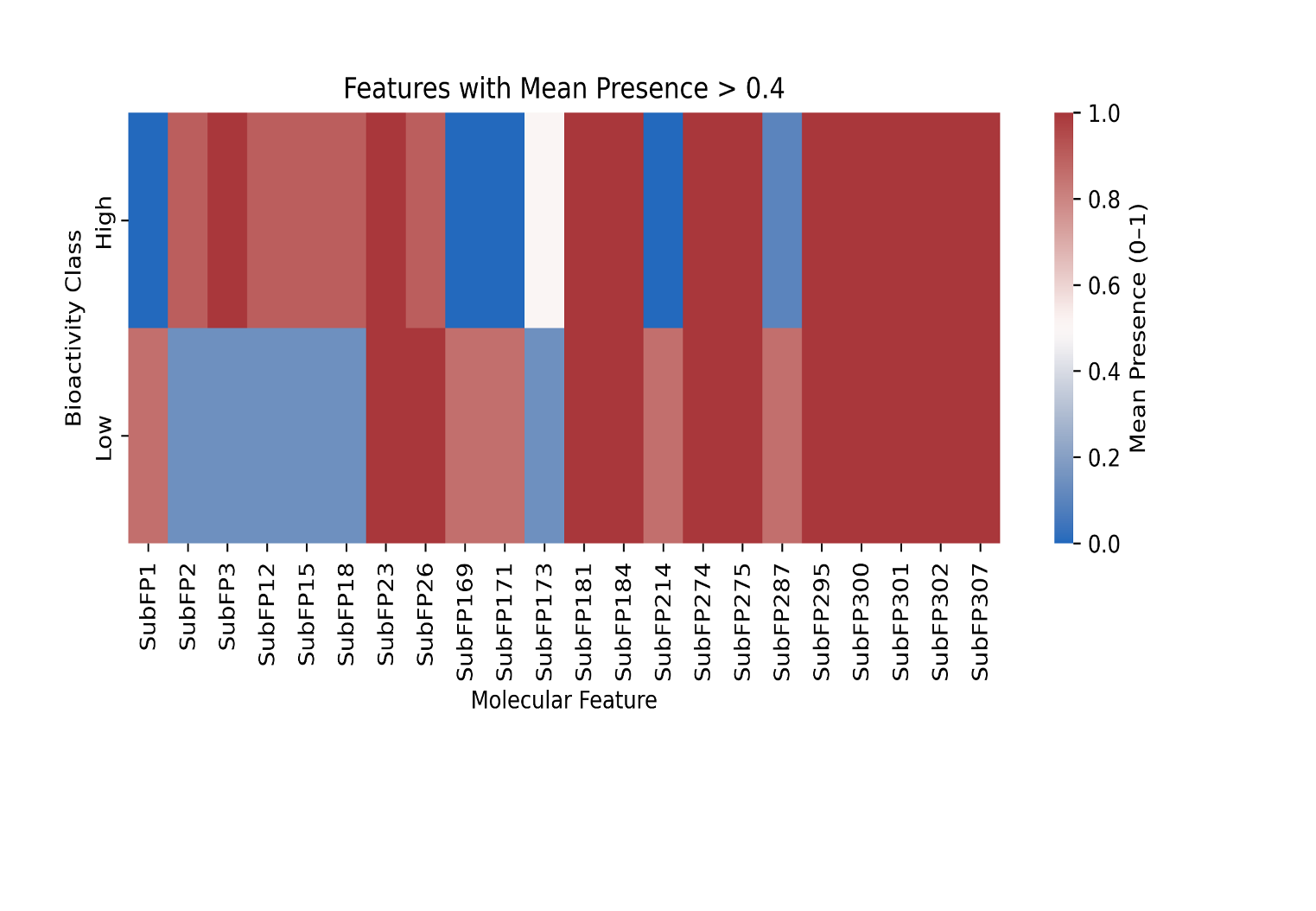
*

***Figure S3****: SAR analysis of 17 previously discovered Mtb ATP synthase inhibitors*

*
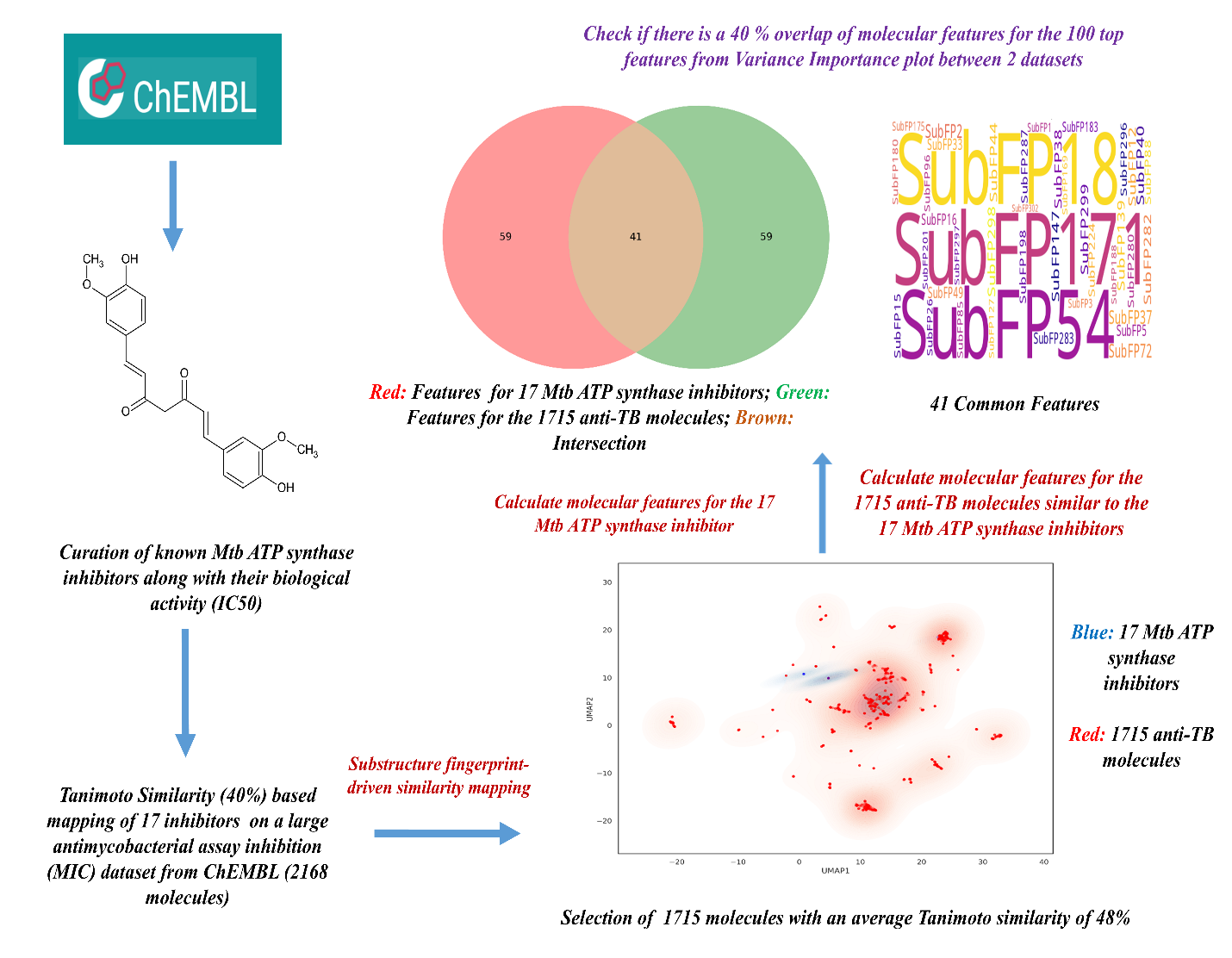
*

***Figure S4****: Chemical space mapping for generation of QSAR dataset*

*
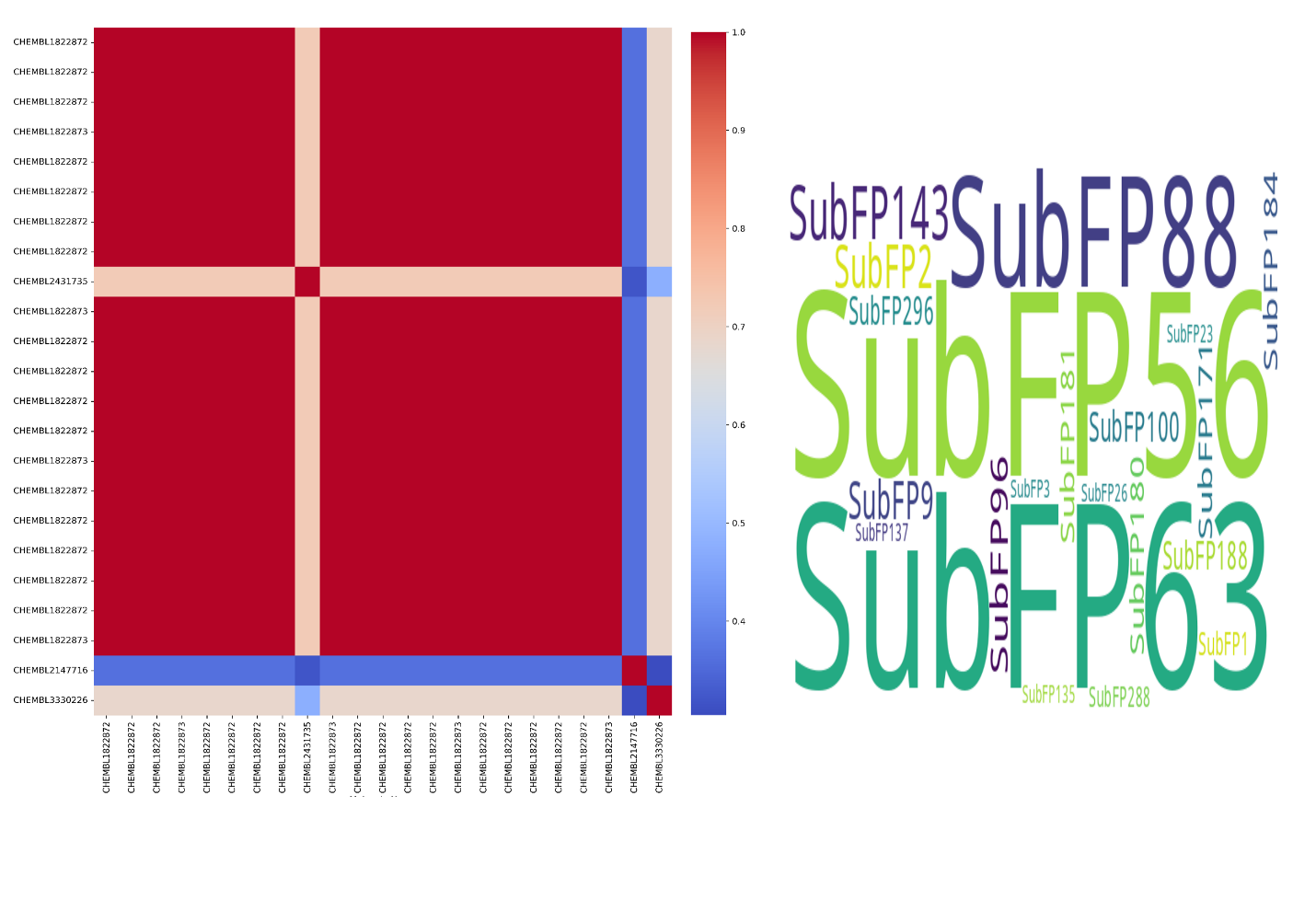
*

***Figure S5****: Molecular similarity analysis of high activity molecules of the training set of ANN-QSAR model*

*
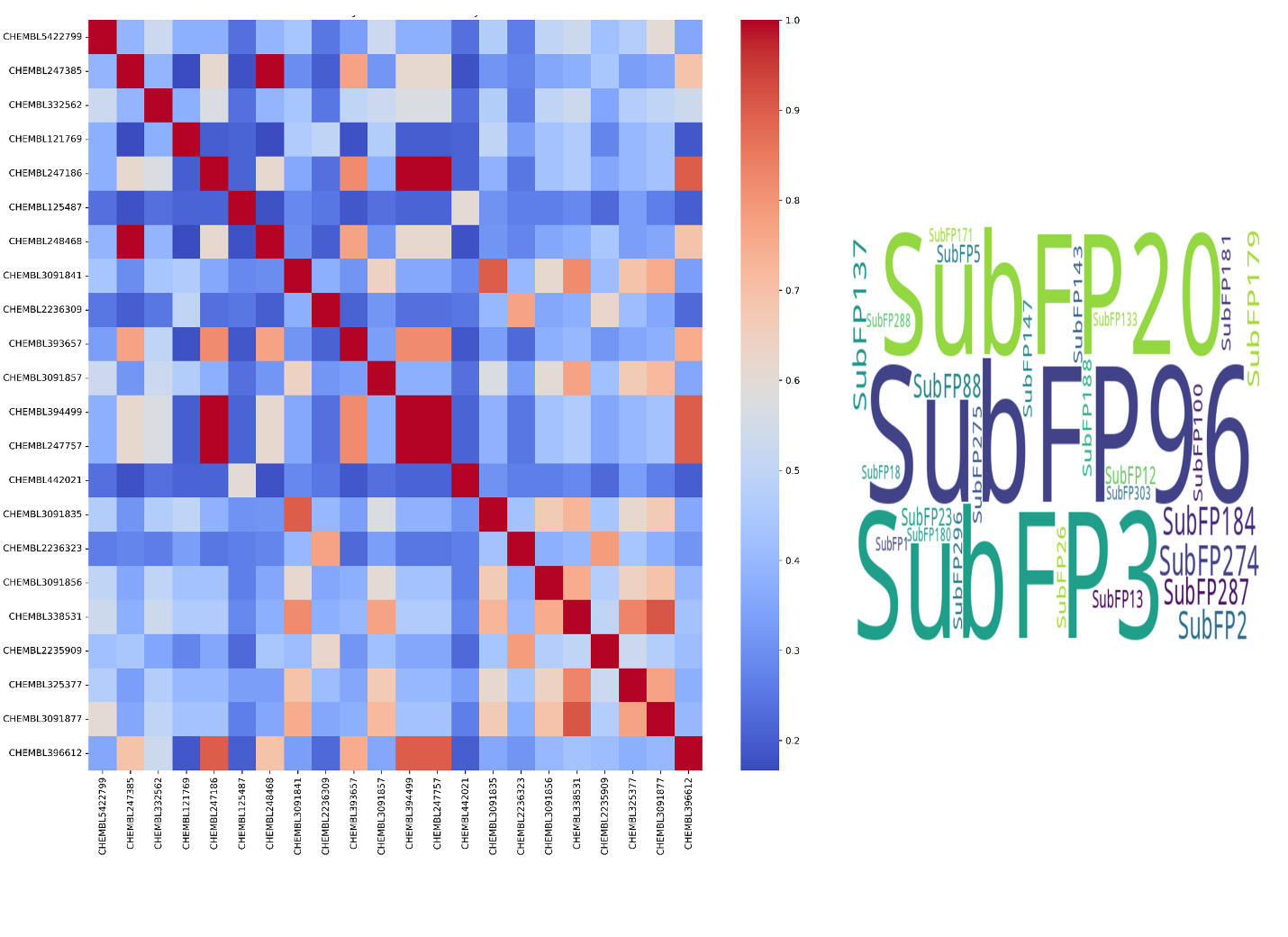
****Figure S6****: Molecular similarity analysis of low activity molecules of the training set of ANN-QSAR model*

*
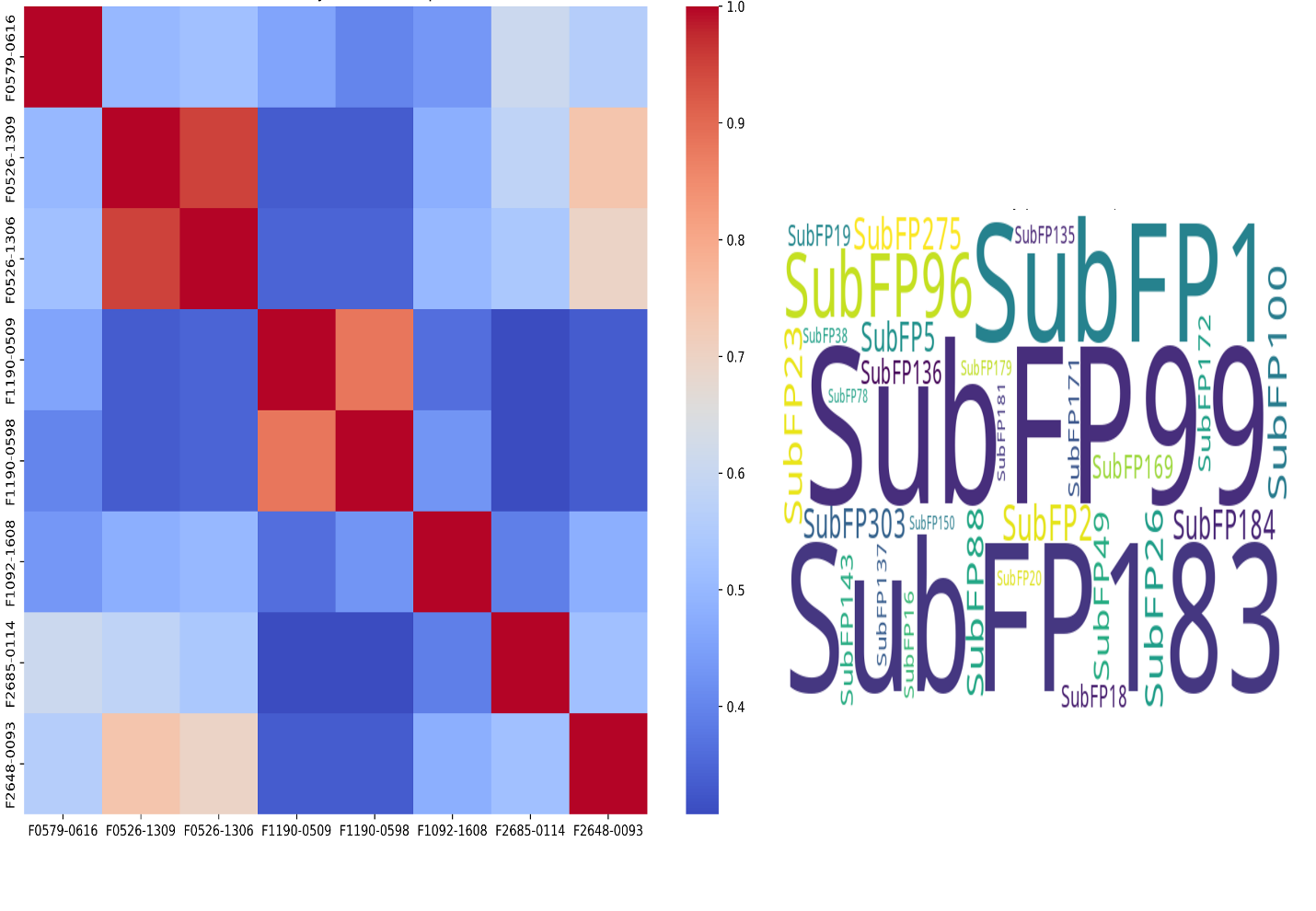
*

***Figure S7****: Molecular similarity analysis of the top 8 computational hits derived from combinatorial pharmacophore and QSAR modeling*

**a**


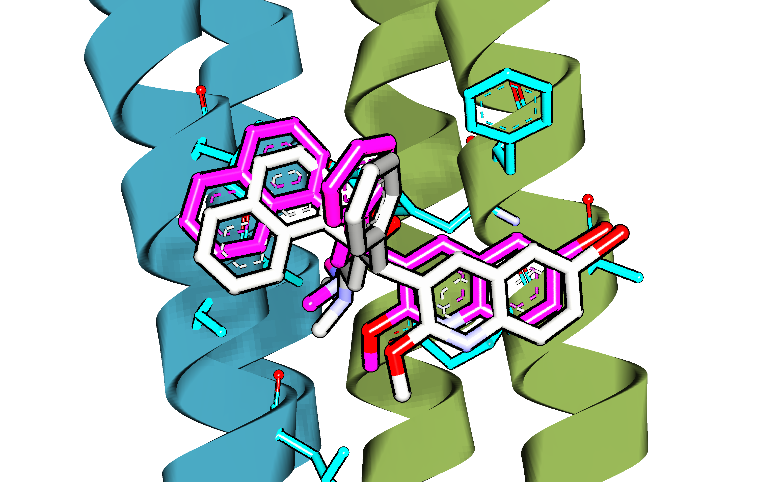

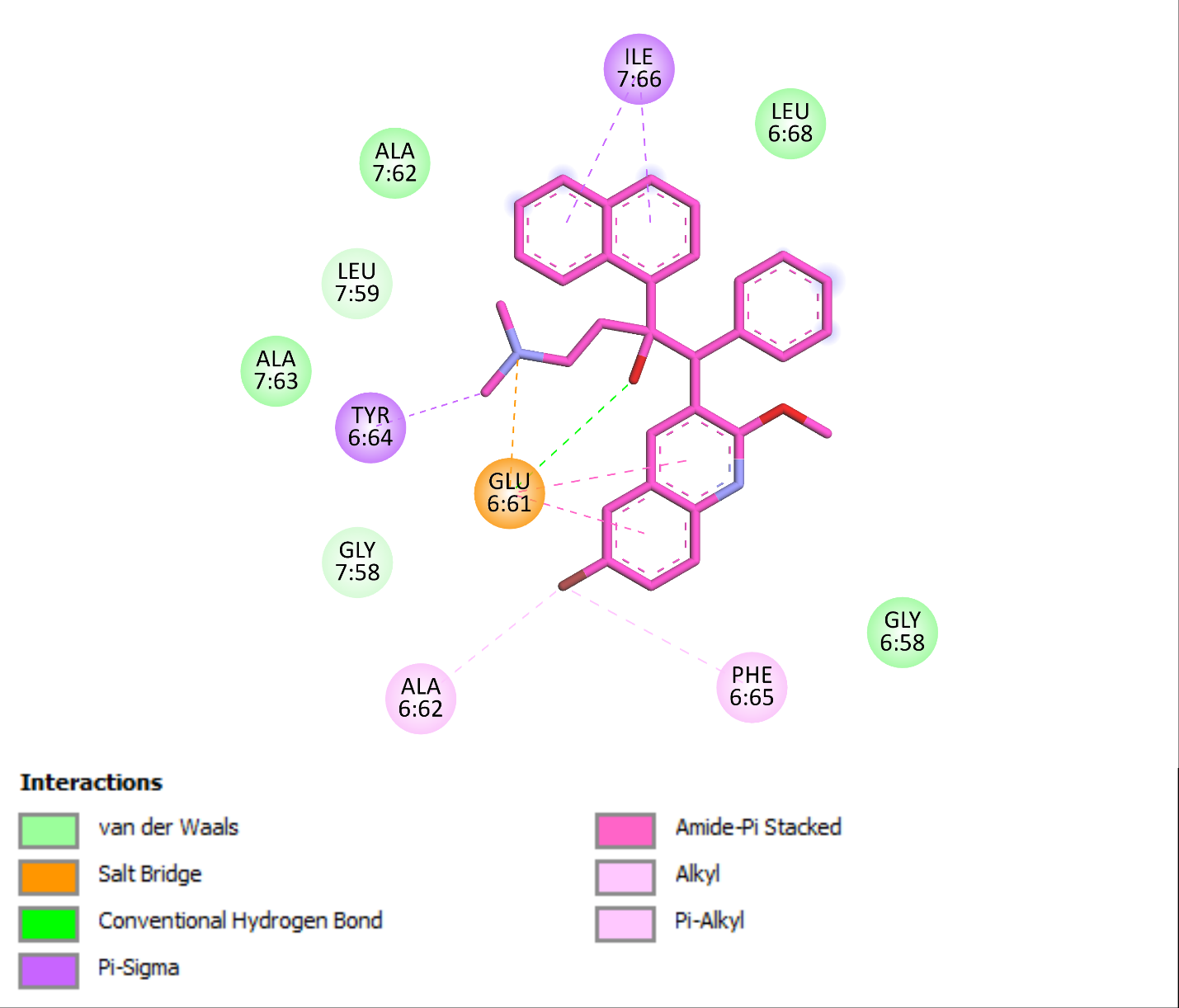


**b**

***Figure S8****: Validation step;(a) 2D interaction diagram of re-docked bedaquiline inside ATP synthase binding pocket, (b) Alignment of the co-crystallized, colored in white, and re-docked, colored in pink, bedaquiline.*


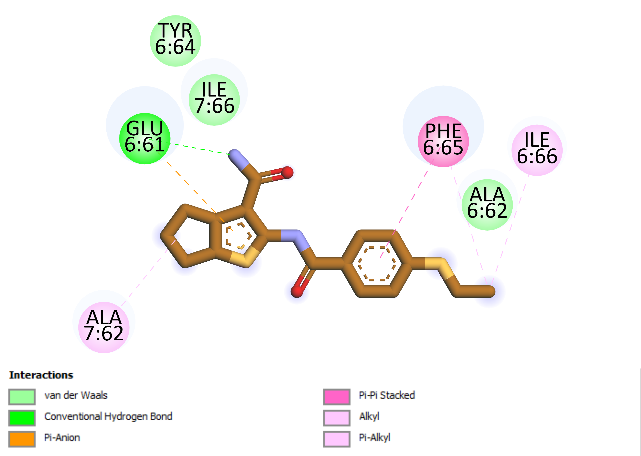

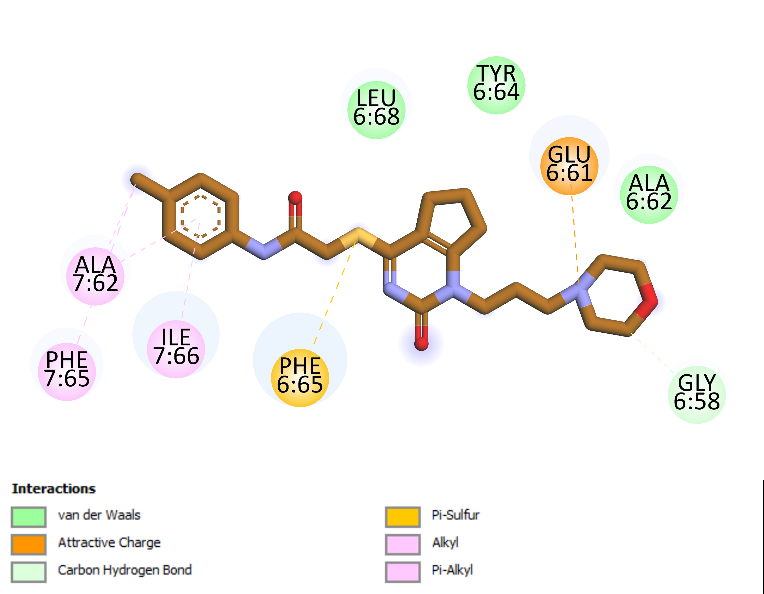

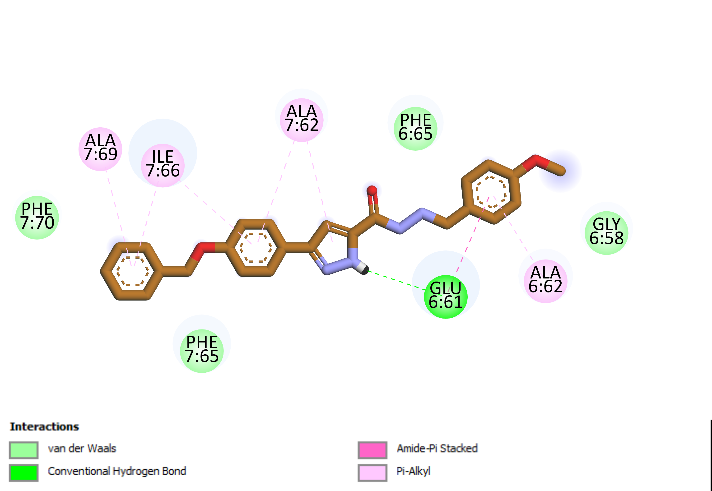

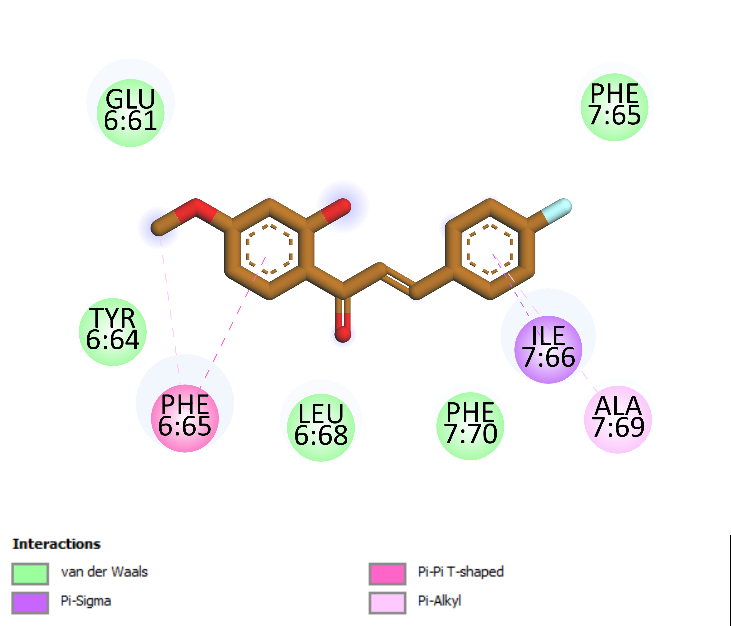

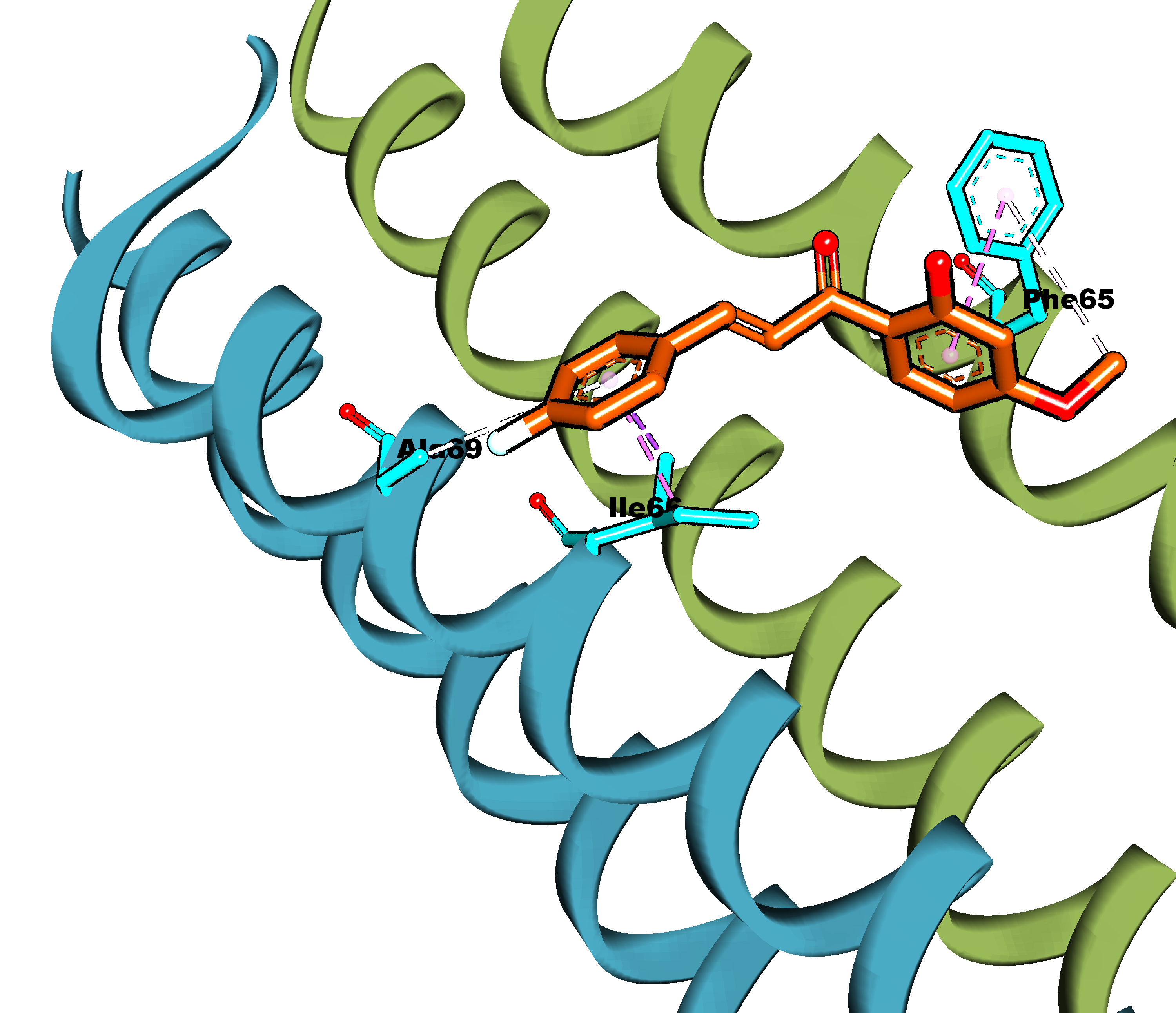

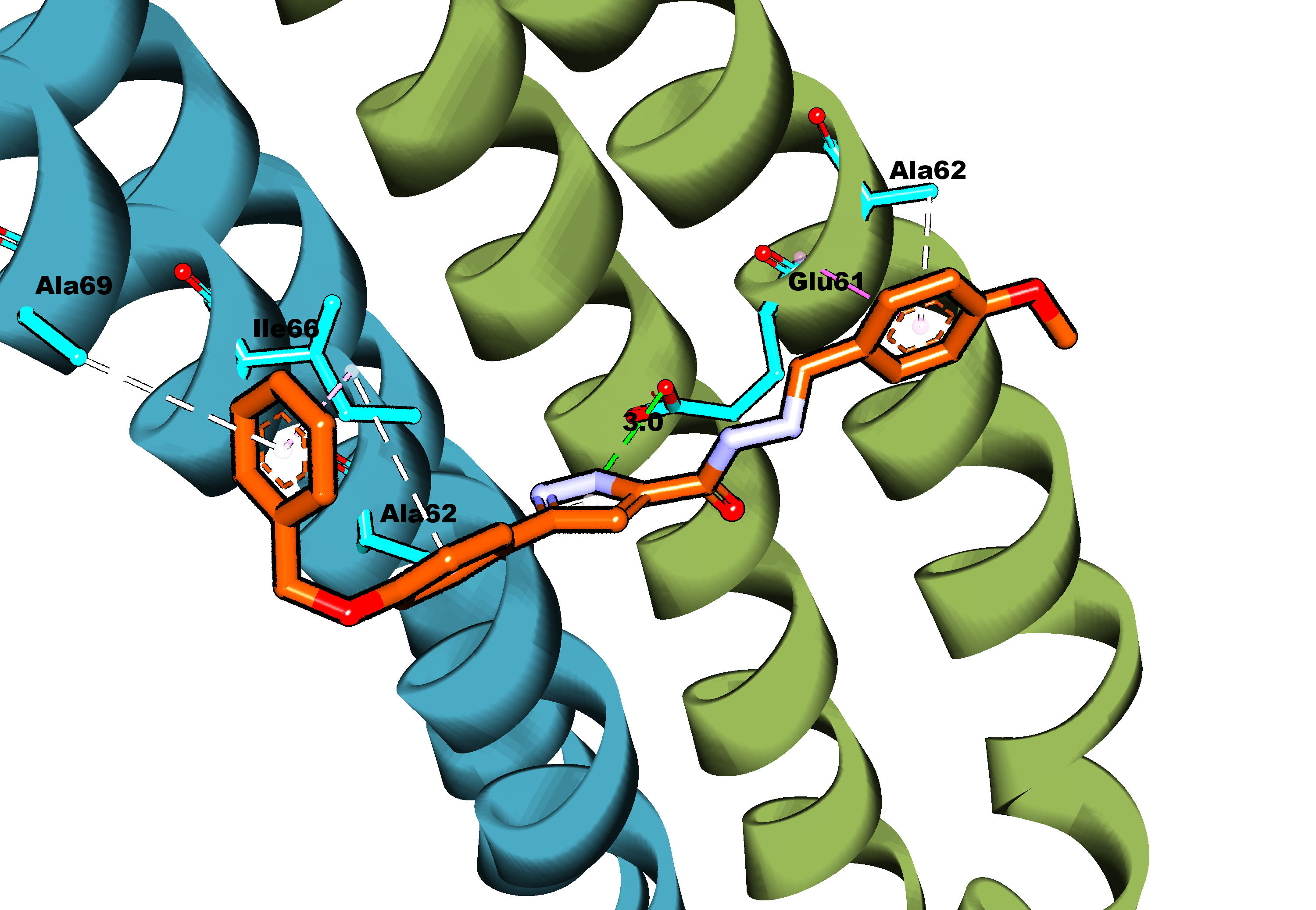

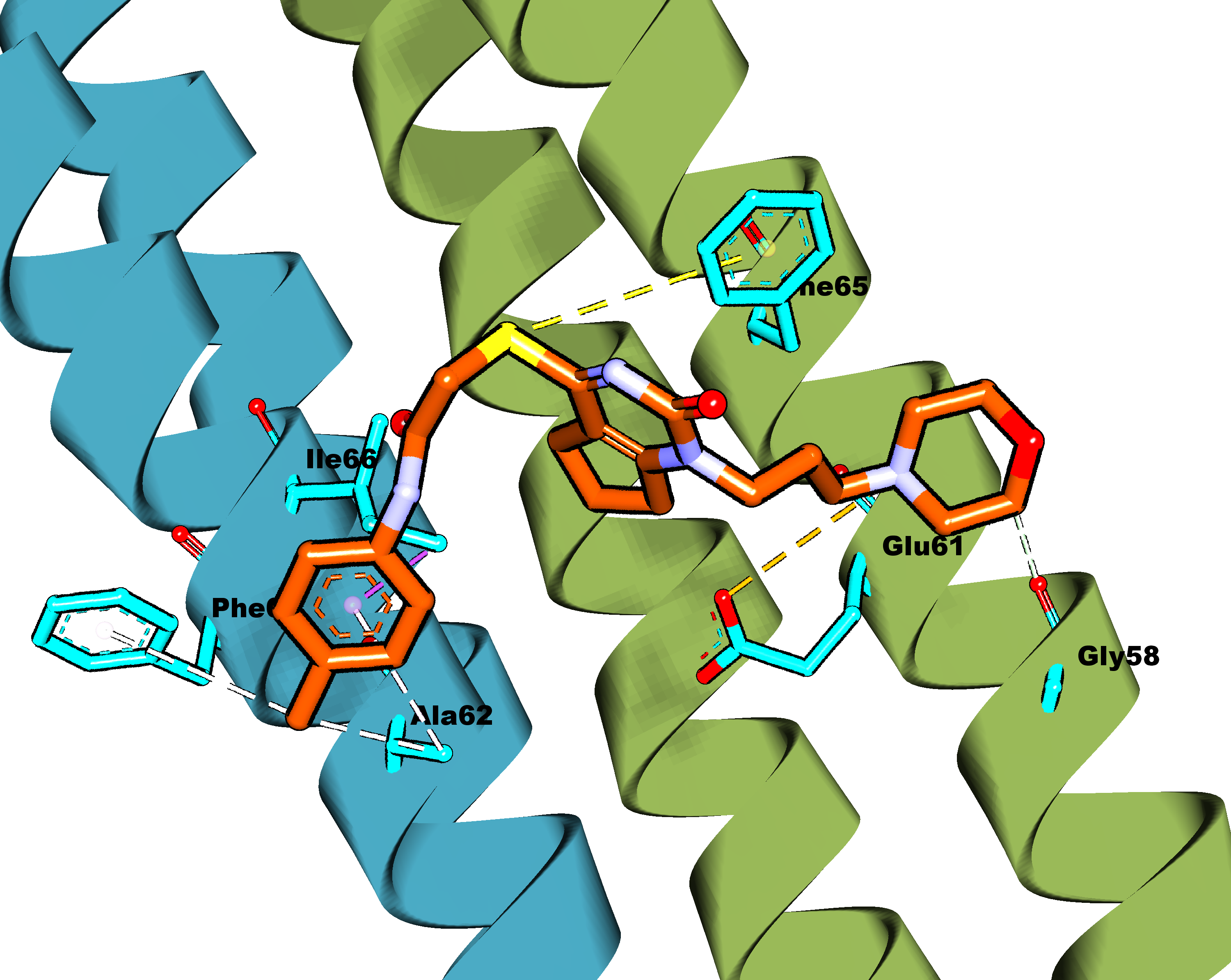

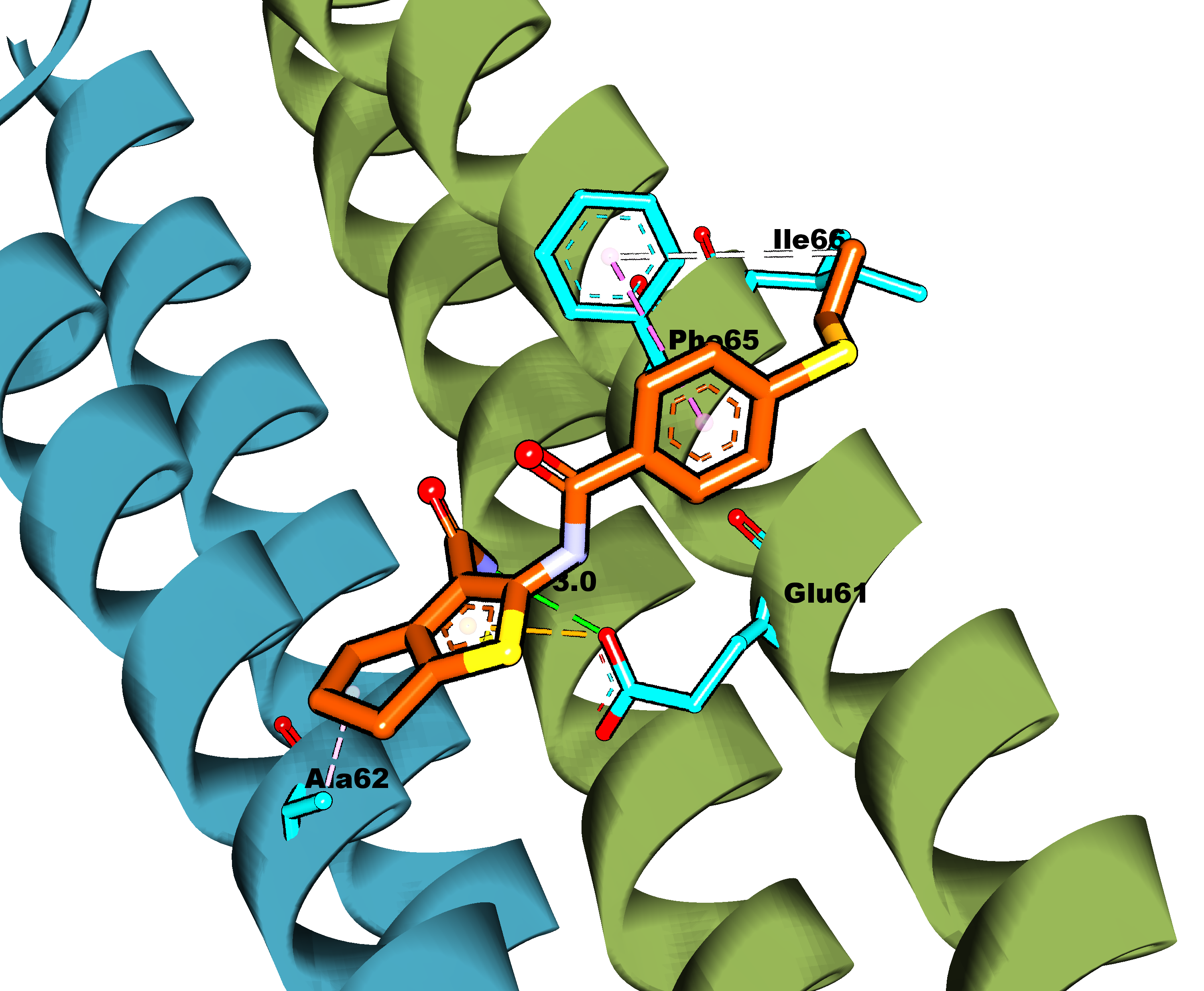


**a**

**b**

**c**

**d**

**Figure S9**: (a) 3D and 2D binding interactions of F1190-0598, (b) 3D and 2D binding interactions of F1092-1608, (c) 3D and 2D binding interactions of F2685-0114, (d) 3D and 2D binding interactions of F2648-0093 within the ATP synthase binding pocket. In 3D view, hits are shown in orange, amino acids in cyan, and the protein backbone in blue and green for chains 6 and 7, respectively.


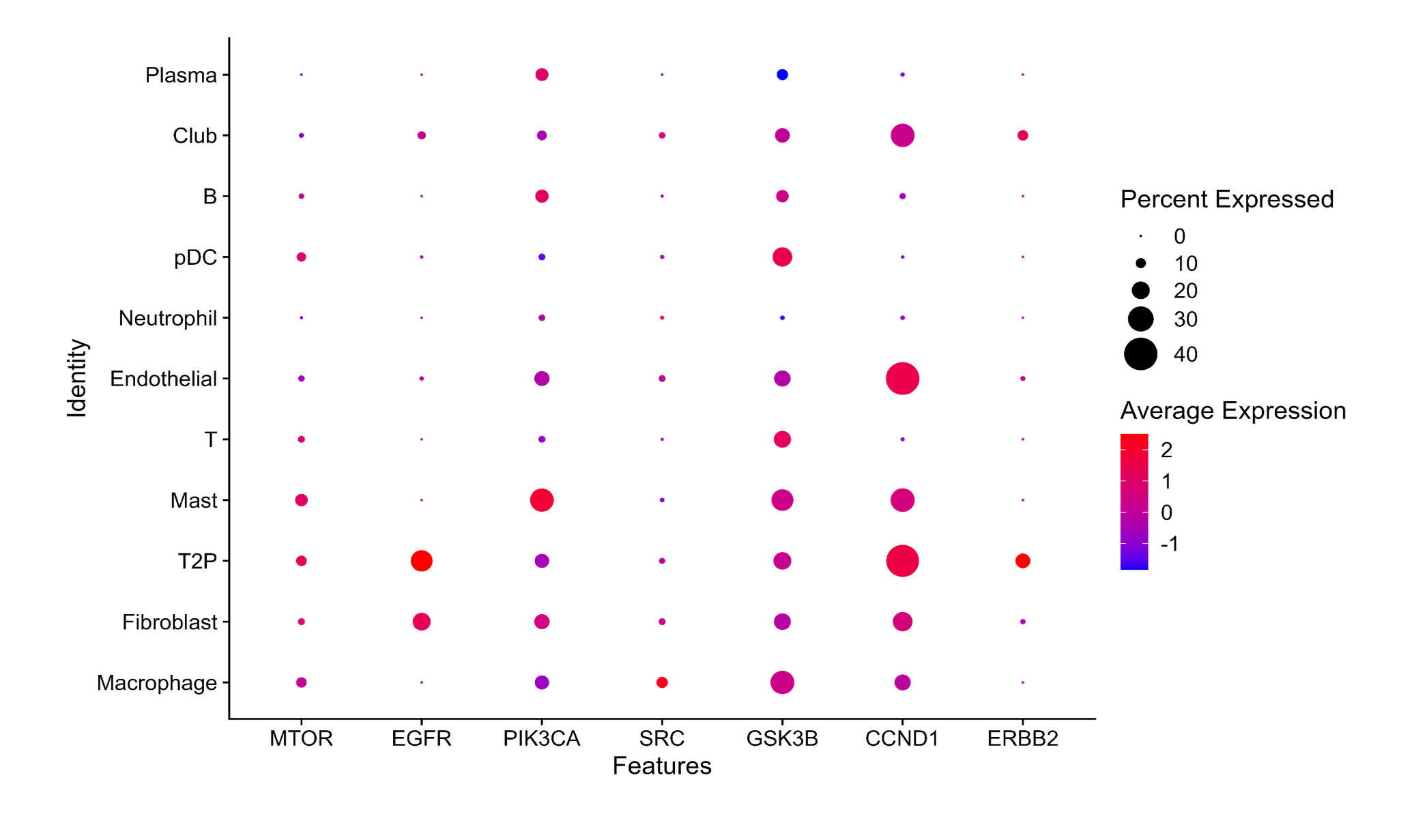


***Figure S10****: ScRNA expression analysis of the top hub genes of molecules F0526-1306 and F0526-1309 through dot plot analysis*

*
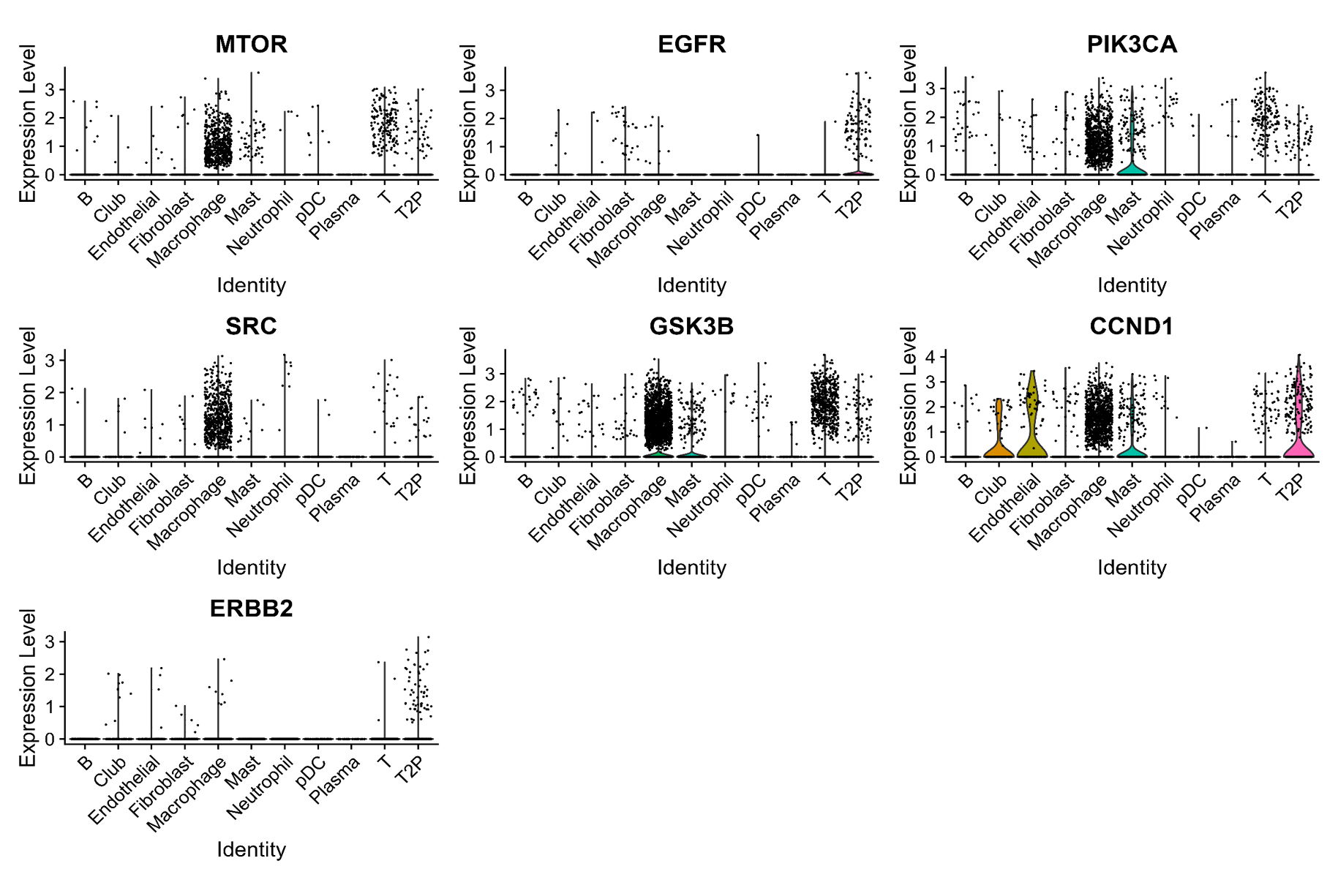
*

***Figure S11****:* *ScRNA expression analysis of the top hub genes of molecules F0526-1306 and F0526-1309 through violin plot analysis*


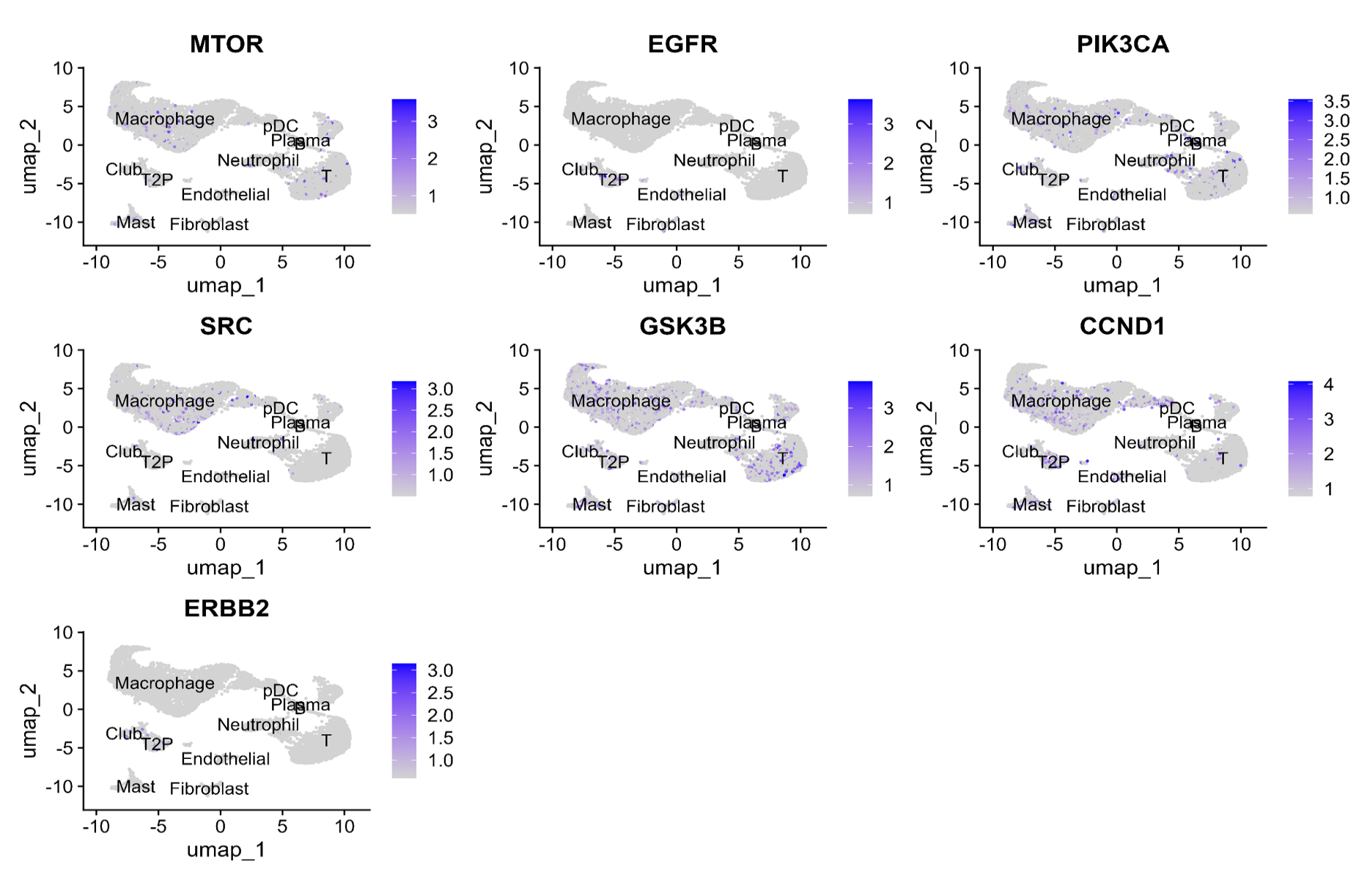


***Figure S12****: ScRNA expression analysis of the top hub genes of molecules F0526-1306 and F0526-1309 through UMAP feature plot analysis*
